# Neighborhood based computational approaches for the prediction of lncRNA-disease associations

**DOI:** 10.1186/s12859-024-05777-8

**Published:** 2024-05-13

**Authors:** Mariella Bonomo, Simona E. Rombo

**Affiliations:** 1 Kazaam Lab s.r.l., Palermo, Italy; 2 Department of Mathematics and Computer Science, University of Palermo, Palermo, Italy

**Keywords:** LncRNA-disease associations, Molecular interactions, Bioinformatics, Long non-coding RNA

## Abstract

**Motivation:**

Long non-coding RNAs (lncRNAs) are a class of molecules involved in important biological processes. Extensive efforts have been provided to get deeper understanding of disease mechanisms at the lncRNA level, guiding towards the detection of biomarkers for disease diagnosis, treatment, prognosis and prevention. Unfortunately, due to costs and time complexity, the number of possible disease-related lncRNAs verified by traditional biological experiments is very limited. Computational approaches for the prediction of disease-lncRNA associations allow to identify the most promising candidates to be verified in laboratory, reducing costs and time consuming.

**Results:**

We propose novel approaches for the prediction of lncRNA-disease associations, all sharing the idea of exploring associations among lncRNAs, other intermediate molecules (e.g., miRNAs) and diseases, suitably represented by tripartite graphs. Indeed, while only a few lncRNA-disease associations are still known, plenty of interactions between lncRNAs and other molecules, as well as associations of the latters with diseases, are available. A first approach presented here, NGH, relies on neighborhood analysis performed on a tripartite graph, built upon lncRNAs, miRNAs and diseases. A second approach (CF) relies on collaborative filtering; a third approach (NGH-CF) is obtained boosting NGH by collaborative filtering. The proposed approaches have been validated on both synthetic and real data, and compared against other methods from the literature. It results that neighborhood analysis allows to outperform competitors, and when it is combined with collaborative filtering the prediction accuracy further improves, scoring a value of AUC equal to 0966.

**Availability:**

Source code and sample datasets are available at: https://github.com/marybonomo/LDAsPredictionApproaches.git

## Introduction

More than $98\%$ of the human genome consists of non-coding regions, considered in the past as “junk” DNA. However, in the last decades evidence has been shown that non-coding genome elements often play an important role in regulating various critical biological processes [1]. An important class of non-coding molecules which have started to receive great attention in the last few years is represented by long non-coding RNAs (lncRNAs), that is, RNAs not translated into functional proteins, and longer than 200 nucleotides.

LncRNAs have been found to interplay with other molecules in order to perform important biological tasks, such as modulating chromatin function, regulating the assembly and function of membraneless nuclear bodies, interfering with signalling pathways [2, 3]. Many of these functions ultimately affect gene expression in diverse biological and physiopathological contexts, such as in neuronal disorders, immune responses and cancer. Therefore, the alteration and dysregulation of lncRNAs have been associated with the occurrence and progress of many complex diseases [4].

The discovery of novel lncRNA-disease associations (LDAs) may provide valuable input to the understanding of disease mechanisms at lncRNA level, as well as to the detection of disease biomarkers for disease diagnosis, treatment, prognosis and prevention. Unfortunately, verifying that a specific lncRNA may have a role in the occurrence/progress of a given disease is an expensive process, therefore the number of disease-related lncRNAs verified by traditional biological experiments is yet very limited. Computational approaches for the prediction of potential LDAs can effectively decrease the time and cost of biological experiments, allowing for the identification of the most promising lncRNA-disease pairs to be further verified in laboratory (see [5] for a comprehensive review on the topic). Such approaches often train predictive models on the basis of the known and experimentally validated lncRNA-disease pairs (e.g., [6–9]). In other cases, they rely on the analysis of lncRNAs related information stored in public databases, such as their interaction with other types of molecules (e.g., [10–15]). As an example, large amounts of lncRNA-miRNA interactions have been collected in public databases, and plenty of experimentally confirmed miRNA-disease associations are available as well. However, although non-coding RNA function and its association with human complex diseases have been widely studied in the literature (see [16–18]), how to provide biologists with more accurate and ready-to-use software tools for LDAs prediction is yet an open challenge, due to the specific characteristics of lncRNAs (e.g., they are much less characterized than other non-coding RNAs.)

**Fig. 1:**
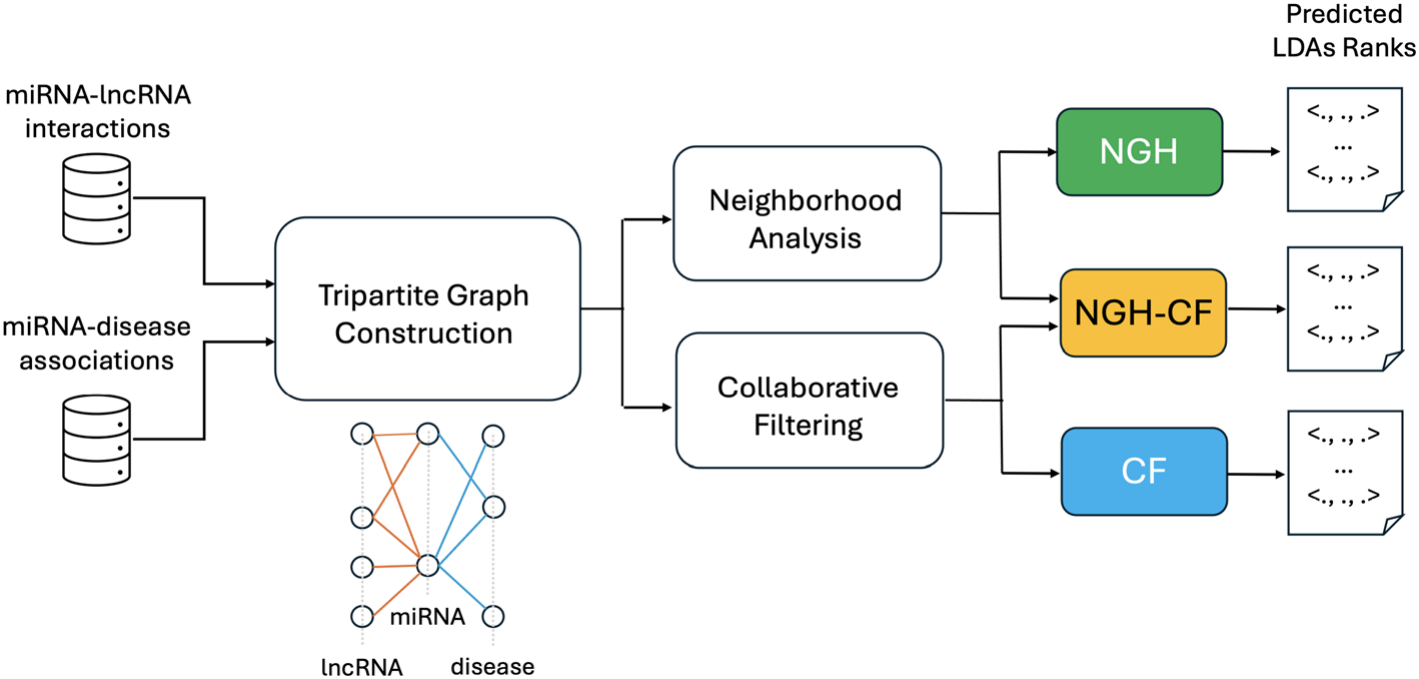
Flowchart of the research pipeline. The miRNA-lncRNA interactions and miRNA-disease associations are exploited for the construction of the tripartite graph. The tripartite graph, in its turn, is at the basis of both neighborhood analysis and collaborative filtering steps, from which the three proposed approaches are obtained: NGH from neighborhood analysis, CF from collaborative filtering, NGH-CF from the combination of the two ones. Each prediction approach returns in output a LDAs rank

We propose three novel computational approaches for the prediction of LDAs, relying on the use of known lncRNA-miRNA interactions (LMIs) and miRNA-disease associations (MDAs). In particular, we model the problem of LDAs prediction as a neighborhood analysis performed on tripartite graphs, where the three sets of vertices represent lncRNAs, miRNAs and diseases, respectively, and vertices are linked according to LMIs and MDAs. Based on the assumption that similar lncRNAs interact with similar diseases [12], the first approach proposed here (NGH) aims at identifying novel LDAs by analyzing the behaviour of lncRNAs which are *neighbors*, in terms of their intermediate relationships with miRNAs. The main idea here is that neighborhood analysis automatically guides towards the detection of similar behaviours, and without the need of using a-priory known LDAs for training. Therefore, differently than other approaches from the literature, those proposed here do not involve verified LDAs in the prediction step, thus avoiding possible biases due to the fact that the number and variety of verified LDAs is yet very limited. The second presented approach (CF) relies on collaborative filtering, applied on the basis of common miRNAs shared by different lncRNAs. We have also explored the combination of neighborhood analysis with collaborative filtering, showing that this notably improves the LDAs prediction accuracy. Indeed, the third approach we have designed (NGH-CF) boosts NGH with collaborative filtering, and it is the best performing one, although also NGH and CF have been able to reach high accuracy values across all the different considered validation tests. In particular, Fig. 1 summarizes the research flowchart explained above.

The proposed approaches have been exhaustively validated on both synthetic and real datasets, and the result is that they outperform (also significantly) the other methods from the literature. The experimental analysis shows that the improvement in accuracy achieved by the methods proposed here is due to their ability in capturing specific situations neglected by competitors. Examples of that are represented by true LDAs, detected by our approaches and not by the other approaches in the literature, where the involved lncRNA does not present intermediate molecules in common with the associated disease, although its *neighbor lncRNAs* share a large number of miRNAs with that disease. Moreover, it is shown that our approaches are robust to noise obtained by perturbing a controlled percentage of lncRNA-miRNA interactions and miRNA-disease associations, with NGH-CF the best one also for robustness. The obtained experimental results show that the prediction methods proposed here may effectively support biologists in selecting significant associations to be further verified in laboratory.

Novel putative LDAs coming from the consensus of the three proposed methods, and not yet registered in the available databases as experimentally verified, are provided. Interestingly, the core of novel LDAs returned with highest score by all three approaches finds evidence in the recent literature, while many other high scored predicted LDAs involve less studied lncRNAs, thus providing useful insights for their better characterization.

## Background

A first group of approaches aim at using existing true validated cases to train the prediction system, in order to make it able to correctly detect novel cases.

In [19] a Laplacian Regularized Least Squares is proposed to infer candidates LDAs (*LRLSLDA*) by applying a semi-supervised learning framework. LRLSLDA assumes that similar diseases tend to correlate with functionally similar lncRNAs, and vice versa. Thus, known LDAs and lncRNA expression profiles are combined to prioritize disease-associated lncRNA candidates by LRLSLDA, which does not require negative samples (i.e., confirmed uncorrelated LDAs). In [20] the method *SKF-LDA* is proposed that constructs a lncRNA-disease correlation matrix, based on the known LDAs. Then, it calculates the similarity between lncRNAs and that between diseases, according to specific metrics, and integrates such data. Finally, a predicted LDA matrix is obtained by the Laplacian Regularized Least Squares method. The method *ENCFLDA* [6] combines matrix decomposition and collaborative filtering. It uses matrix factorization combined with elastic networks and a collaborative filtering algorithm, making the prediction model more stable and eliminating the problem of data over-fitting. *HGNNLDA* recently proposed in [21] is based on hypergraph neural network, where the associations are modeled as a lncRNA-drug bipartite graph to build lncRNA hypergraph and drug hypergraph. Hypergraph convolution is then used to learn correlation of higher-order neighbors from the lncRNA and drug hypergraphs. *LDAI-ISPS* proposed in [22] is a LDAs inference approach based on space projections of integrated networks, recostructing the disease (lncRNA) integrated similarities network via integrating multiple information, such as disease semantic similarities, lncRNA functional similarities, and known LDAs. A space projection score is finally obtained via vector projections of the weighted networks. In [7] a consensual prediction approach called *HOPEXGB* is presented, to identify disease-related miRNAs and lncRNAs by high-order proximity preserved embedding and extreme gradient boosting. The authors build a heterogeneous disease-miRNA-lncRNA (DML) information network by linking lncRNA, miRNA, and disease nodes based on their correlation, and generate a negative dataset based on the similarities between unknown and known associations, in order to reduce the false negative rate in the data set for model construction. The method *MAGCNSE* proposed in [23] builds multiple feature matrices based on semantic similarity and disease Gaussian interaction profile kernel similarity of both lncRNAs and diseases. MAGCNSE adaptively assigns weights to the different feature matrices built upon the lncRNAs and diseases similarities. Then, it uses a convolutional neural network to further extract features from multi-channel feature matrices, in order to obtain the final representations of lncRNAs and diseases that is used for the LDAs prediction task.

*LDAFGAN* [8] is a model designed for predicting associations between long non-coding RNAs (lncRNAs) and diseases. This method is based on a generative and a discriminative networks, typically implemented as multilayer fully connected neural networks, which generate synthetic data based on some underlying distribution. The generative and discriminative networks are trained together in an adversarial manner. The generative network tries to generate realistic representations of lncRNA-disease associations, while the discriminative network tries to distinguish between real and fake associations. This adversarial training process helps the generative network learn to generate more realistic associations. Once the model is trained, it can predict associations between new lncRNAs and diseases without requiring associated data for those specific lncRNAs. The model captures the data distribution during training, which enables it to make predictions even for unseen lncRNAs. The approach *GCNFORMER* [9] is based on graph convolutional network and transformer. First, it integrates the intraclass similarity and interclass connections between miRNAs, lncRNAs and diseases, building a graph adjacency matrix. Then, the method extracts the features between various nodes, by a graph convolutional network. To obtain the global dependencies between inputs and outputs, a transformer encoder with a multiheaded attention mechanism to forecast lncRNA-disease associations is finally applied.

As for the approaches summarized above, it is worth to point out that they may suffer of the fact that the experimentally verified LDAs are still very limited, therefore the training set may be rather incomplete and not enough diversified. For this reason, when such approaches are applied for de novo LDAs prediction, their performance may drastically go down [12].

Other approaches from the literature use intermediate molecules (e.g., miRNA) to infer novel LDAs. Such approaches are the most related to those we propose here.

The author in [11] proposes *HGLDA*, relying on HyperGeometric distribution for LDAs inference, that integrates MDAs and LMIs information. HGLDA has been successfully applied to predict Breast Cancer, Lung Cancer and Colorectal Cancer-related lncRNAs. *NcPred* [10] is a resource propagation technique, using a tripartite network where the edges associate each lncRNA with a disease through its targets. The algorithm proposed in [10] is based on a multilevel resource transfer technique, which computes the weights between each lncRNA-disease pair and, at each step, considers the resource transferred from the previous step. The approach in [24], referred to as *LDA-TG* for short in the following, is the antecedent of the approaches proposed here. It relies on the construction of a tripartite graph, built upon MDAs and LMIs. A score is assigned to each possible LDA (*l*, *d*) by considering both their respective interactions with common miRNAs, and the interactions with miRNAs shared by the considered disease *d* and other lncRNAs in the neighborhood of *l* on the tripartite graph. The approaches proposed here differ from LDA-TG for two main reasons. First, the score of LDA-TG is different from the one we introduce here, that allows to reach a better accuracy. Second, a further step based on collaborative filtering is considered here, which also improves the accuracy performance. A method for LDAs prediction relying on a matrix completion technique inspired by recommender systems is presented in [14]. A two-layer multi-weighted nearest-neighbor prediction model is adopted, using a method similar to memory-based collaborative filtering. Weights are assigned to neighbors for reassigning values to the target matrix, that is an adjacency matrix consisting of lncRNAs, diseases and miRNA. *SSMF-BLNP* [25] is based on the combination of selective similarity matrix fusion (SSMF) and bidirectional linear neighborhood label propagation (BLNP). In SSMF, self-similarity networks of lncRNAs and diseases are obtained by selective preprocessing and nonlinear iterative fusion. In BLNP, the initial LDAs are employed in both lncRNA and disease directions as label information for linear neighborhood label propagation.

A third category includes approaches based on integrative frameworks, proposed to take into account different types of information related to lncRNAs, such as their interactions with other molecules, their involvement in disorders and diseases, their similarities. This may improve the prediction step, taking into account simultaneously independent factors.

*IntNetLncSim* [26] relies on the construction of an integrated network that comprises lncRNA regulatory data, miRNA-mRNA and mRNA-mRNA interactions. The method computes a similarity score for all pairs of lncRNAs in the integrated network, then analyzes the information flow based on random walk with damping. This allows to infer novel LDAs by exploring the function of lncRNAs. *SIMCLDA* [12] identifies LDAs by using inductive matrix completion, based on the integration of known LDAs, disease-gene interactions and gene-gene interactions. The main idea in [12] is to extract feature vectors of lncRNAs and diseases by principal component analysis, and to calculate the interaction profile for a new lncRNA by the interaction profiles. *MFLDA* [27] is a Matrix Factorization based LDAs prediction model that first encodes directly (or indirectly) relevant data sources related to lncRNAs or diseases in individual relational data matrices, and presets weights for these matrices. Then, it simultaneously optimizes the weights and low-rank matrix tri-factorization of each relational data matrix. *RWSF-BLP*, proposed in [28], applies a random walk-based multi-similarity fusion method to integrate different similarity matrices, mainly based on semantic and expression data, and bidirectional label propagation. The framework *LRWRHLDA* is proposed in [15] based on the construction of a global multi-layer network for LDAs prediction. First, four isomorphic networks including a lncRNA similarity network, a disease similarity network, a gene similarity network and a miRNA similarity network are constructed. Then, six heterogeneous networks involving known lncRNA-disease, lncRNA-gene, lncRNA-miRNA, disease-gene, disease-miRNA, and gene-miRNA associations are built to design the multi-layer network. In [29] the *LDAP-WMPS* LDA prediction model is proposed, based on weight matrix and projection score. LDAP-WMPS consists on three steps: the first one computes the disease projection score; the second step calculates the lncRNA projection score; the third step fuses the disease projection score and the lncRNA projection score proportionally, then it normalizes them to get the prediction score matrix.

For most of the approaches summarized above, the performance is evaluated using the LOOCV framework, such that each known LDA is left out in turn as a test sample, and how well this test sample is ranked relative to the candidate samples (all the LDAs without the evidence to confirm their relationships) is computed.

## Methods

The main goal of the research presented here is to provide more accurate computational methods for the prediction of novel LDAs, candidate for experimental validation in laboratory. To this aim, external information on both molecular interactions (e.g., lncRNA-miRNA interactions) and genotype-phenotype associations (e.g., miRNA-disease associations) is assumed to be available. Indeed, while only a restricted number of validated LDAs is yet available, large amounts of interactions between lncRNAs and other molecules (e.g., miRNAs, genes, proteins), as well as associations between these other molecules and diseases, are known and annotated in curated databases.

A commonly recognized assumption is that lncRNAs with similar behaviour in terms of their molecular interactions with other molecules, may also reflect such a similarity for their involvement in the occurrence and progress of disorders and diseases [12]. This is even more effective if the correlation with diseases is “mediated” by the molecules they interact with. Based on this observation, we have designed three novel prediction methods that all consider the notion of lncRNA “neighbors”, intended as lncRNAs which share common mediators among the molecules they physically interact with. Here, we focus on miRNAs as mediator molecules. However, the proposed approaches are general enough to allow also the inclusion of other different molecules. Relationships among lncRNAs, mediators and diseases are modeled through tripartite graphs in all the proposed approaches (see Fig. 1 that illustrates the flowchart of the presented research pipeline).

*Problem statement* Let ${\mathcal {L}}=\{l_1, l_2, \ldots , l_h\}$ be a set of lncRNAs and ${\mathcal {D}}=\{d_1, d_2, \ldots , d_k\}$ be a set of diseases. The goal is to return an ordered set of triplets ${\mathcal {R}}=\{\langle l_x, d_y, s_{xy}\rangle \}$ (with $x\in [1,h]$, and $y\in [1,k]$), ranked according to the score $s_{xy}$.

The top triplets in ${\mathcal {R}}$ correspond to those pairs $(l_x, d_y)$ with most chances to represent putative LDAs which may be considered for further analysis in laboratory, while the triplets in the bottom correspond to lncRNAs and diseases which are unlikely to be related each other. A key aspect for the solution of the problem defined above is the score computation, that is the main aim of the approaches introduced in the following.

### NGH: neighborhood based approach

A model of tripartite graph is adopted here to take into account that lncRNAs interacting with common mediators may be involved in common diseases.

Let $T_{LMD}=\langle I, A \rangle$ be a tripartite graph defined on the three sets of disjoint vertexes *L*, *M* and *D*, such that $(l,m) \in I$ are edges between vertexes $l \in L$ and $m \in M$, $(m,d) \in A$ are edges between vertexes $m \in M$ and $d \in D$, respectively. In particular, *L* is associated to a set of lncRNAs, *M* to a set of miRNA and *D* to a set of diseases. Moreover, edges of the type (*l*, *m*) represent molecular interactions between lncRNAs and miRNA, experimentally validated in laboratory; edges of the type (*m*, *d*) correspond to known miRNA-disease associations, according to the existing literature. In both cases, interactions and associations annotated and stored in public databases may be taken into account.

The following definitions hold.

#### Definition 1

*(Neighbors)* Two lncRNAs $l_h, l_k \in L$ are *neighbors* in $T_{LMD}=\langle I, A \rangle$ if there exists at least a $m_x \in M$ such that $(l_h, m_x) \in I$ and $(l_k, m_x) \in I$.

#### Definition 2

*(Prediction Score)* The *Prediction Score* for the pair $(l_i,d_j)$ such that $l_i \in L$ and $d_j \in D$ is defined as:

1 $$\begin{aligned} s_{ij} = \alpha \cdot \frac{|M_{l_i}\bigcap M_{d_j}|}{|M_{l_i} \bigcup M_{d_j}|}+(1-\alpha )\cdot \frac{ |\bigcup _x (M_{l_x}\bigcap M_{d_j})|}{|\bigcup _x (M_{l_x} \bigcup M_{d_j})|} \end{aligned}$$

where:

- $M_{l_i}$ is the set of annotated miRNA interacting with $l_i$,
- $M_{d_j}$ is the set of miRNA found to be associated to $d_j$,
- $M_{l_x}$ is the set of miRNA interacting with the neighbor $l_x$ of $l_i$ (for each neighbor of $l_i$),
- $\alpha$ is a real value in [0, 1] used to balance the two terms of the formula.

#### Definition 3

*(Normalized prediction score)* The *Normalized Prediction Score* for the pair $(l_i,d_j)$ such that $l_i \in L$, $d_j \in D$ and $s_{ij}$ is the Prediction Score for $(l_i,d_j)$, is defined as:

2 $$\begin{aligned} {\hat{s}}_{ij}=\frac{s_{ij}}{\sum _{hk}s_{hk}}, \forall h \in [1, \ldots , |L|], \forall k\in [1, \ldots , |D|] \end{aligned}$$

### NGH-CF: NGH extended with collaborative filtering

We remark that the main idea here is trying to infer the behaviour of a lncRNA, from that of its neighbors. Moreover, it is worth to point out that the notion of neighbor is related to the presence of miRNAs interacting with the same lncRNAs. However, not all the miRNA-lncRNA interactions have already been discovered, and miRNA-disease associations as well. This intuitively reminds to a typical context of data incompleteness where Collaborative Filtering may be successful in supporting the prediction process [30].

In more detail, what to be encoded by the Collaborative Filter is that lncRNAs presenting similar behaviours in terms of interactions with miRNAs, should reflect such a similarity also in their involvement with the occurrence and progress of diseases, mediated by those miRNAs. To this aim, a matrix *R* is considered here such that each element $r_{ij}$ represents if (or to what extent) the lncRNA *i* and the disease *j* may be considered related. We call *R* *relationship matrix* (it is also known as *rating matrix* in other contexts, such as for example in the prediction of user-item associations). How to obtain $r_{ij}$ is at the basis of the two variants of the approach presented in this section.

Due to the fact that *R* is usually a very sparse matrix, it can be factored into other two matrices *L* and *D* such that *R* $\approx$ $L$ ^T^ $D$. In particular, matrix factorization models map both lncRNAs and diseases to a joint latent factor space *F* of dimensionality *f*, such that each lncRNA *i* is associated with a vector $l_i \in F$, each disease *j* with a vector $d_j \in F$, and their relationships are modeled as inner products in that space. Indeed, for each lncRNA *i*, the elements of $l_i$ measure the extent to which it possesses those latent factors, and the same holds for each disease *j* and the corresponding elements of $d_j$. The resulting dot product in the factor space captures the affinity between lncRNA *i* and disease *j*, with reference to the considered latent factors. To this aim, there are two important tasks to be solved:

1. Mapping lncRNAs and diseases into the corresponding latent factors vectors.
2. Fill the matrix *R*, that is, the training set.

To learn the factor vectors $l_i$ and $d_j$, a possible choice is to minimize the regularized squared error on the set of known relationships:

$$\sum _{(i,j) \in \chi } (r_{ij} - l_i^Td_j)^2,$$

where $\chi$ is the set of (*i*, *j*) pairs for which $r_{ij}$ is not equal to zero in the matrix *R*. To this aim, we apply the ALS technique [31], which rotates between fixing the $l_i$’s and fixing the $d_j$’s. When all $l_i$’s are fixed, the system recomputes the $d_j$’s by solving a least-squares problem, and vice versa.

Filling the matrix *R* is performed according to two different criteria, resulting in the two different variants of the approach presented in this section, namely, CF and NGH-CF, respectively. According to the first criteria (CF), $r_{ij}$ is set equal to 1 if the lncRNA *i* and the disease *j* share at least one miRNA in common, to 0 otherwise. The second variant (NGH-CF) works instead as a booster to improve the accuracy of NGH. In this latter case, the matrix *R* is filled by the normalized score (2). For both variants, the considered score to rank the predicted LDAs is given by the final value returned by the ALS technique applied on the corresponding matrix *R*.

### Validation methodologies

We remark that the proposed approaches for LDAs prediction return a rank of LDAs, sorted according to the score that is characteristic of the considered approach, such that top triplets may be assumed as the most promising putative LDAs for further analysis in laboratory. As in other contexts [19–33], the *performance* of a prediction tool may be evaluated using suitable *external criteria*. Here, an external criterion relies on the existence of LDAs that are known to be true from the literature or, even better, from public repositories, where associations already verified in laboratory are annotated. A *gold standard* is constructed, containing only such true LDAs. The putative LDAs returned by the prediction method can thus be compared against those in the gold standard. In order to work properly, this validation methodology requires the gold standard information to be *independent* on that considered, in its turn, from the method under evaluation during its prediction task. This is satisfied in our case, due to the fact that all three approaches introduced in the previous sections do not exploit any type of knowledge referred to known LDAs during prediction, relying instead on known miRNA-lncRNA interactions and miRNA-disease associations, which come from independent sources.

According to the above mentioned validation methodology, the proposed approaches can be validated with references to the Receiver Operating Characteristics (ROC) analysis [34]. In particular, each predicted LDA is associated to a label, that is *true* if that association is contained in the considered gold standard, and *false* otherwise.

By varying the threshold value, it is possible to compute the true positive rate (TPR) and the false positive rate (FPR), by refferring to the percentage of the true/false predictions whose ranking is higher/below than the considered threshold value. ROC curve can be drawn by plotting TPR versus FPR at different threshold values. The Area Under ROC Curve (ROC-AUC) is further calculated to evaluate the performance of the tested methods. ROC-AUC equal to 1 indicates perfect performance, ROC-AUC equal to 0.5 random performance.

Similarly to the ROC curve, the Precision-Recall (PR) curve can be drawn as well, combining the positive predictive value (PPV, Precision), i.e., the fraction of predicted LDAs which are true in the gold standard, and the TPR (Recall), in a single visualization, at the threshold varying. The higher on y-axis the obtained curve is, the better the prediction method performance. The Area Under PR curve (AUPR) is more sensitive than AUC to the improvements for the positive class prediction [35], that is important for the case studied here. Indeed, only true LDAs are known, therefore no negative samples are included in the gold standard.

Another important measure useful to evaluate the prediction accuracy of a method and that can be considered here is the F1-score, defined as the harmonic mean of Precision and Recall to symmetrically represent both metrics in a single one.

## Results

### Datasets

We have validated the proposed approaches on both syntetic and real datasets, as explained below.

#### Synthetic data

A synthetic dataset has been built with 15 lncRNAs, 35 miRNA and 10 diseases, such that three different sets of LDAs may be identified, as follows (see also Table 1, where the characteristics of each LDA are summarized).

- *Set 1:* 26 LDAs, such that each lncRNA has from 3 to 4 miRNAs shared with the same disease *(strongly linked lncRNAs)*.
- *Set 2:* 16 LDAs, each lncRNA having only one miRNA shared with a disease, and from 2 to 5 neighbors that are strongly linked with that same disease *(directly linked lncRNAs and strong neighborhood)*.
- *Set 3:* 12 LDAs involving lncRNAs without any miRNA in common with a certain disease, and a number between 2 and 5 neighbors that are strongly linked with that same disease *(only strong neighborhood)*.

**Table 1:** Summary of synthetic data characteristics

LDA			Neighbours	
lncRNA	Disease	N. miRNAs	N. lncRNAs	N. miRNAs
*Set 1*				
l1	d1	3	3	3
l1	d2	3	4	10
l1	d3	3	3	6
l1	d4	3	4	7
l10	d6	3	3	2
l11	d2	3	5	10
l13	d3	3	4	5
l13	d4	4	7	7
l2	d1	3	4	4
l2	d2	3	4	9
l2	d3	3	5	5
l2	d4	4	3	4
l3	d1	3	4	6
l3	d2	3	5	11
l3	d4	3	5	5
l5	d1	3	4	1
l6	d6	3	6	6
l6	d8	3	4	3
l6	d9	4	6	7
l7	d7	3	6	7
l7	d8	3	5	4
l7	d9	3	4	3
l8	d6	4	5	5
l8	d7	4	4	4
l8	d8	3	3	2
l8	d9	3	5	6
*Set 2*				
l10	d10	1	4	7
l10	d7	1	5	6
l12	d2	1	3	10
l12	d3	1	2	9
l13	d2	1	4	8
l15	d5	1	3	7
l14	d2	1	4	11
l14	d4	1	4	9
l4	d1	1	2	8
l4	d2	1	4	12
l4	d4	1	4	8
l9	d6	1	3	9
l9	d7	1	4	10
l9	d9	1	3	9
l3	d3	1	4	9
l6	d7	1	5	8
*Set 3*				
l11	d1	0	3	7
l11	d3	0	2	8
l11	d4	0	3	10
l12	d1	0	2	7
l12	d4	0	4	8
l13	d1	0	3	8
l15	d1	0	5	9
l15	d2	0	3	15
l5	d2	0	4	13
l5	d3	0	3	7
l5	d4	0	2	9
l7	d6	0	3	7
*Others*				
l1	d5	1	1	2
l14	d5	1	2	1
l15	d5	1	1	2
l15	d8	1	1	1
l9	d3	1	1	1
l9	d8	1	1	1
l9	d10	1	1	2
l10	d5	1	2	2
l10	d8	1	1	1
l15	d4	2	1	2
l8	d5	1	2	2
l3	d6	2	1	1
l3	d8	2	1	1
l7	d2	1	2	1
l5	d10	1	2	3
l11	d10	1	1	2
l11	d3	1	1	2
l11	d8	2	2	2
l13	d7	1	2	1
l13	d5	2	2	3
l3	d4	1	1	2
l15	d6	1	1	2
l12	d9	1	2	1

In the first three columns information on the LDA is reported: lncRNA, disease and the number of miRNA shared between them, respectively

Fourth and fifth columns show information on the neighbours of the lncRNA in the first column which share some miRNA with the disease in the second column. In particular, column 4 shows the number of such neighbours, while column 5 the number of miRNAs they share with the disease

#### Real data

Experimentally verified data downloaded from starBase [36] and from HMDD [37] have been considered for the lncRNA-miRNA interactions and for the miRNA-disease associations, respectively. In particular, the latest version of HMDD, updated at 2019, has been used. Overall, $1,\!114$ lncRNAs, $1,\!058$ miRNAs, 885 diseases, $10,\!112$ lncRNA-miRNA interactions and $16,\!904$ miRNA-disease associations have been included in the analysis.

In order to evaluate the prediction accuracy of the approaches proposed here against those from the literature, three different gold standards have been considered. A first gold standard dataset **GS1** has been obtained from the LncRNA-Disease database [38], resulting in 183 known and verified LDAs. A second, more restrictive, gold standard **GS2** with 157 LDAs has been built by the intersection of data from [38] and [39]. Finally, also a larger gold standard dataset **GS3** has been included in the analysis, by extracting LDAs from MNDRv2.0 database [40], where associations both experimentally verified and retrieved from manual literature curation are stored, resulting in 408 known LDAs.

### Comparison on real data

The approaches proposed here have been compared against other approaches from the literature, over the three different gold standards described in the previous Section. In particular, all approaches considered from the literature have been run according to the default setting of their parameters, reported on the corresponding scientific publications and/or on their manual instructions.

**Fig. 2:**
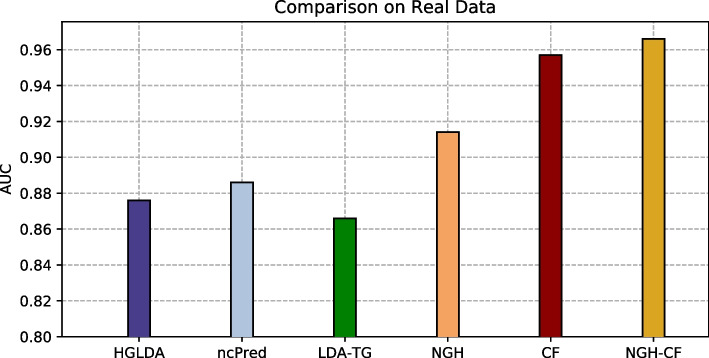
Comparison of the scored AUC on GS1

**Fig. 3:**
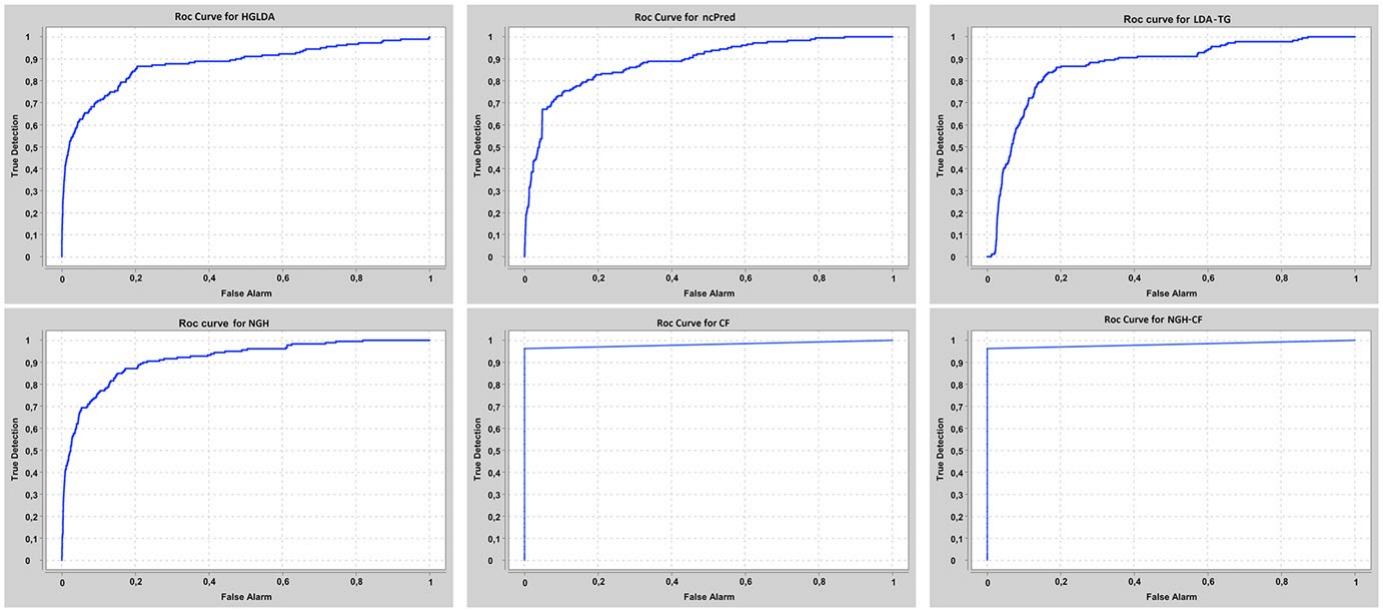
ROC curves for the compared methods on GS1

Our approaches have been compared at first on GS1 against those approaches taking exactly the same input than ours, that are HGLDA [11], ncPred [10] and LDA-TG [24]. In particular, we have implemented HGLDA and used the corresponding p-value score, corrected by FDR as suggested by [11], for the ROC analysis. Moreover, we have normalized also the scores returned by ncPred and LDA-TG for the predicted LDAs, according to the formula in Definition 3. Indeed, we have observed experimentally that such a normalization improves the accuracy of both methods from the literature, resulting in a better AUC. As for the novel approaches proposed here, the Normalized Prediction Score has been considered for NGH, while the approximated rating score resulting from ALS [31] is used for both CF and NGH-CF. Figure 2 shows the AUC scored by each method on GS1, while in Fig. 3 the different ROC curves are plotted. In particular, NGH scores a value of AUC equal to 0.914, thus outperforming the other three methods previously presented in the literature, i.e., HGLDA, ncPred and LDA-TG, that reach 0.876, 0.886 and 0.866, respectively (we remark also that performance of both ncPred and LDA-TG has been slightly improved with respect to their original one, by normalizing their scores). As for the novel approaches based on collaborative filtering, they both present a better accuracy than the others, with CF having AUC equal to 0.957 and NGH-CF to 0.966, respectively. Therefore, these results confirm that taking into account the collaborative effects of lncRNAs and miRNAs is useful to improve LDAs prediction, and the most successful approach is NGH-CF, that is, the neighborhood based approach boosted by collaborative filtering.

Another interesting issue is represented by the “agreement” between the different methods taking the same input, in terms of the returned best scoring LDAs. Table 2 shows the Jaccard Index computed between the proposed approaches and those receiving the same input, on the top $5\%$ LDAs in the corresponding ranks, sorted from the best to the worst score values for each method. It emerges that results by HGLDA and ncPred have a small match with the other approaches (at most 0.23), while NGH-CF has high agreement with CF (0.74), as well as with NGH and LDA-TG (both 0.70). LDA-TG and CF present a sufficient match in their best predictions (0.59). This latter comparison based on agreement shows that approaches based on neighborhood analysis share a larger set of LDAs, in the top part of their ranks.

**Table 2:** Jaccard Index on the top $5\%$ LDAs, for each pair of methods

	HGLDA	ncPred	LDA-TG	NGH	CF	NGH-CF
HGLDA	1	0.23	0.20	0.20	0.15	0.21
ncPred	0.23	1	0.11	0.11	0.10	0.11
LDA-TG	0.20	0.11	1	0.70	0.59	0.70
NGH	0.20	0.11	0.70	1	0.59	0.70
CF	0.15	0.10	0.59	0.59	1	0.74
NGH-CF	0.21	0.11	0.70	0.70	0.74	1

**Fig. 4:**
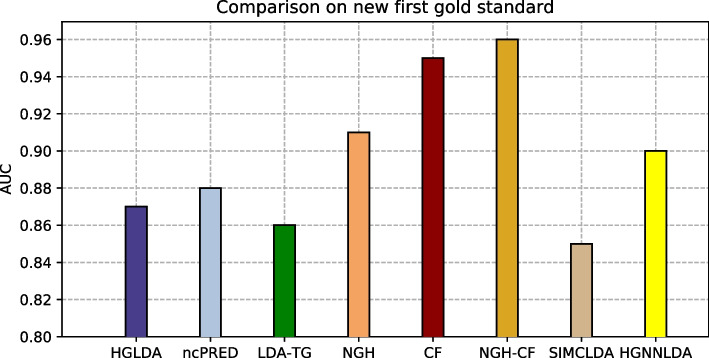
Comparison of the scored AUC on GS2

**Fig. 5:**
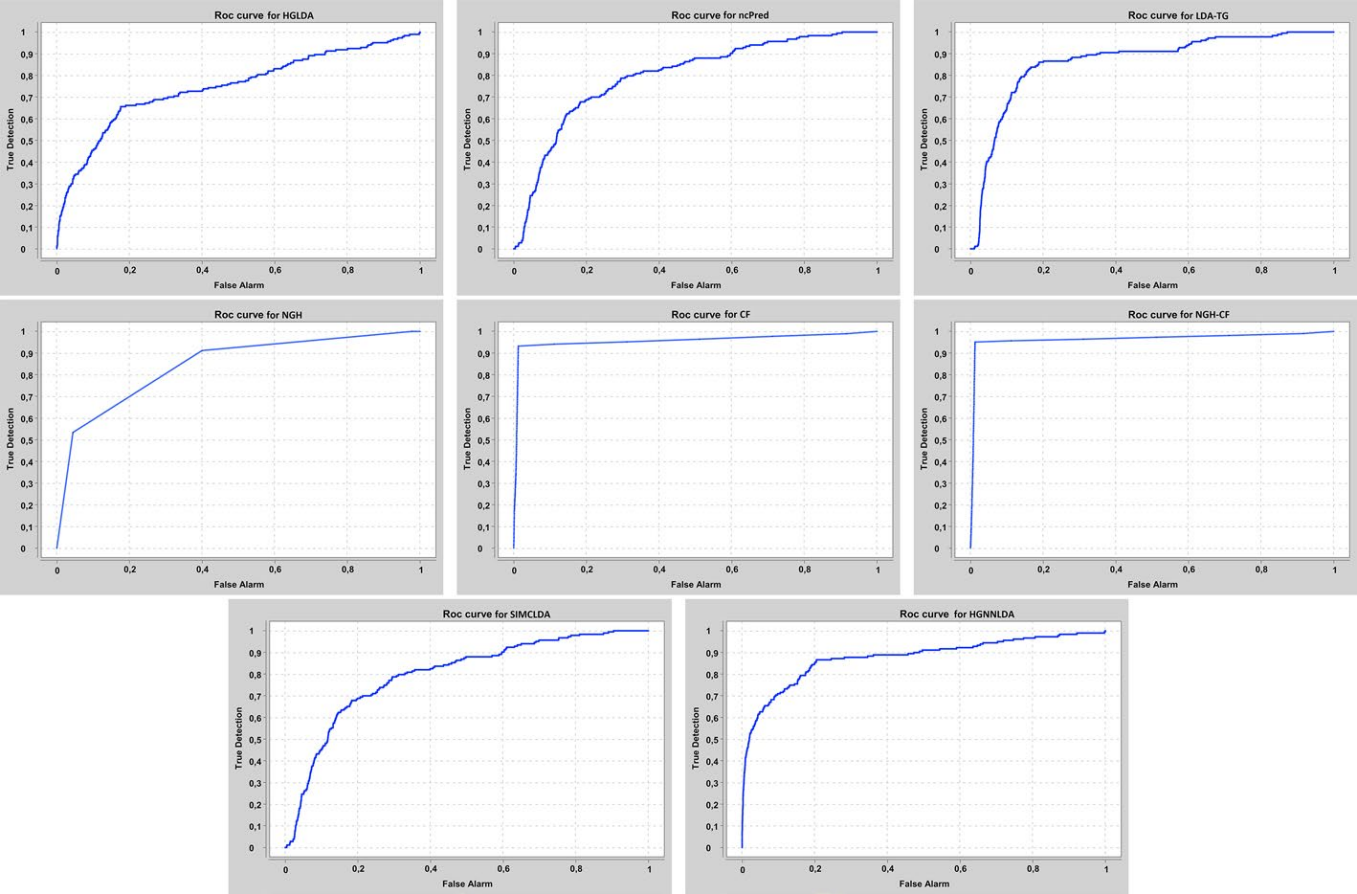
ROC curves for the compared methods on GS2

The proposed approaches have been compared also against other two recent methods from the literature, i.e., SIMCLDA and HGNNLDA, which receive in input different data than ours, including mRNA and drugs. For this reason, the more restrictive gold standard GS2 has been exploited for the comparison, where only lncRNAs and diseases having some correspondences with the additional input data of SIMCLDA and HGNNLDA are included. Figure 4 shows the comparison of the scored AUC on GS2, while Fig. 5 the corresponding ROC curves. In particular, the behaviour of all approaches previously tested does not change significantly on this other gold standard, moreover all the other approaches overcome SIMCLDA. On the other hand, HGNNLDA has a better performance than HGLDA, NcPred and LDA-TG, although it has a worse accuracy than NGH, CF and NGH-CF. The former confirms its superiority with regards to all considered approaches.

**Fig. 6:**
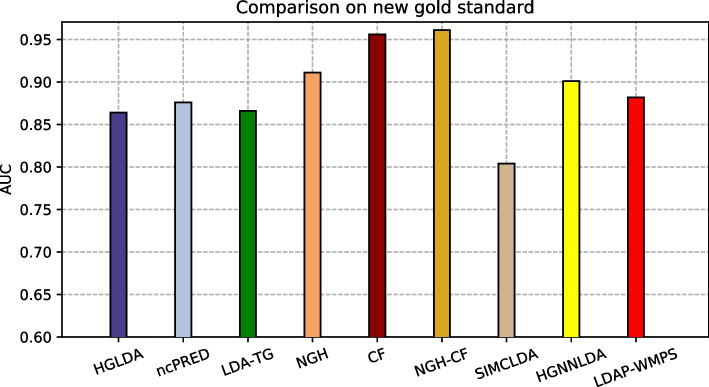
Comparison of the scored AUC on GS3

**Fig. 7:**
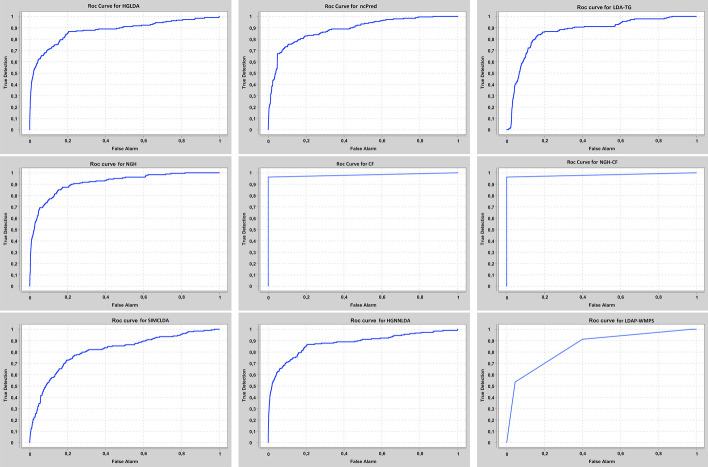
ROC curves for the compared methods on GS3

The proposed approaches have been compared also against LDAP-WMPS on GS3. Figure 6 shows the AUC values scored by all compared approaches on GS3, while Fig. 7 the corresponding ROC curves. In particular, the behaviour of all approaches previously tested does not change on this other gold standard, and LDAP-WMPS has better performance than the other approaches except for NGH, CF, NGH-CF and HGNNLDA.

**Fig. 8:**
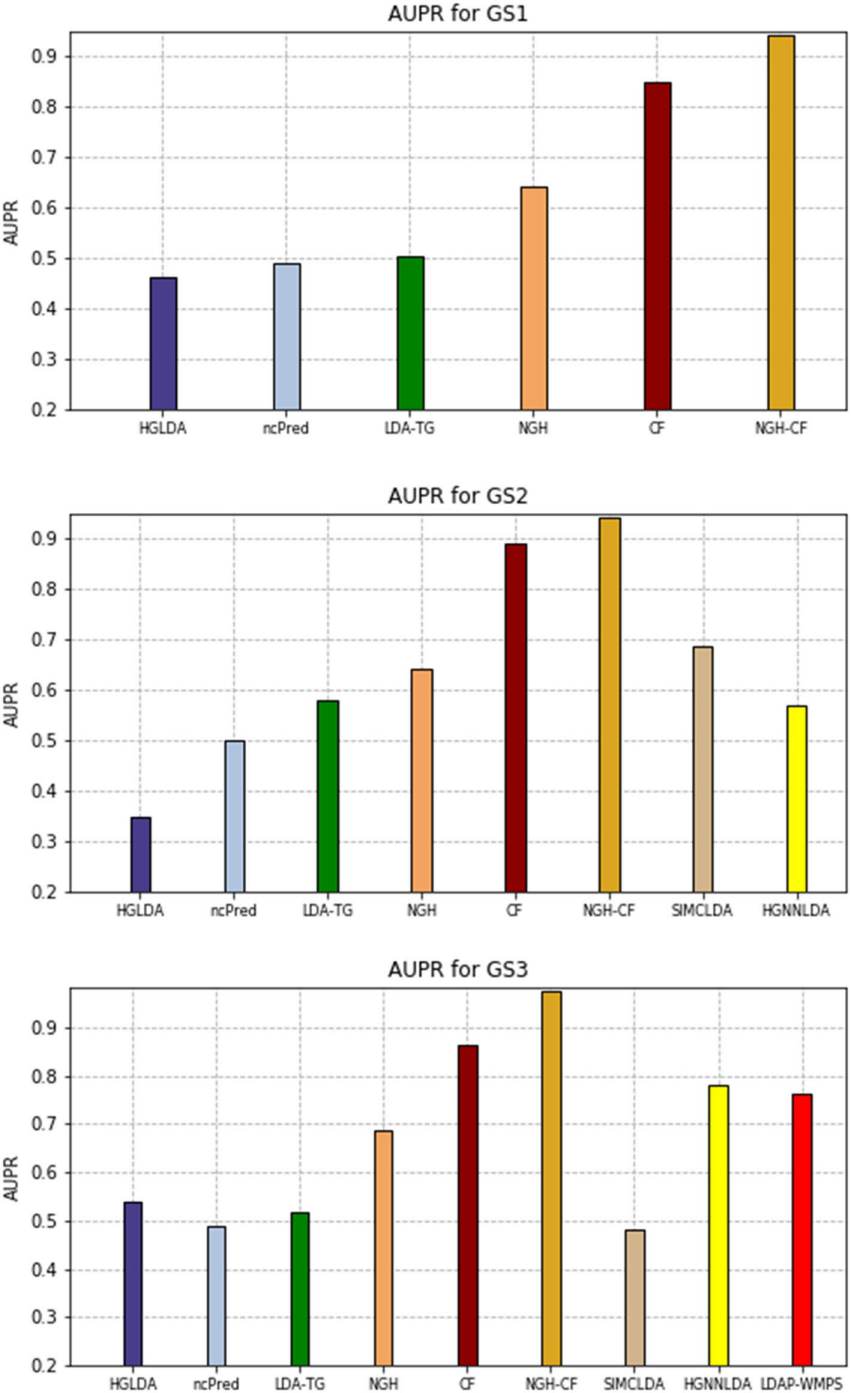
AUPR hystogram for the compared methods on GS1, GS2, GS3

**Fig. 9:**
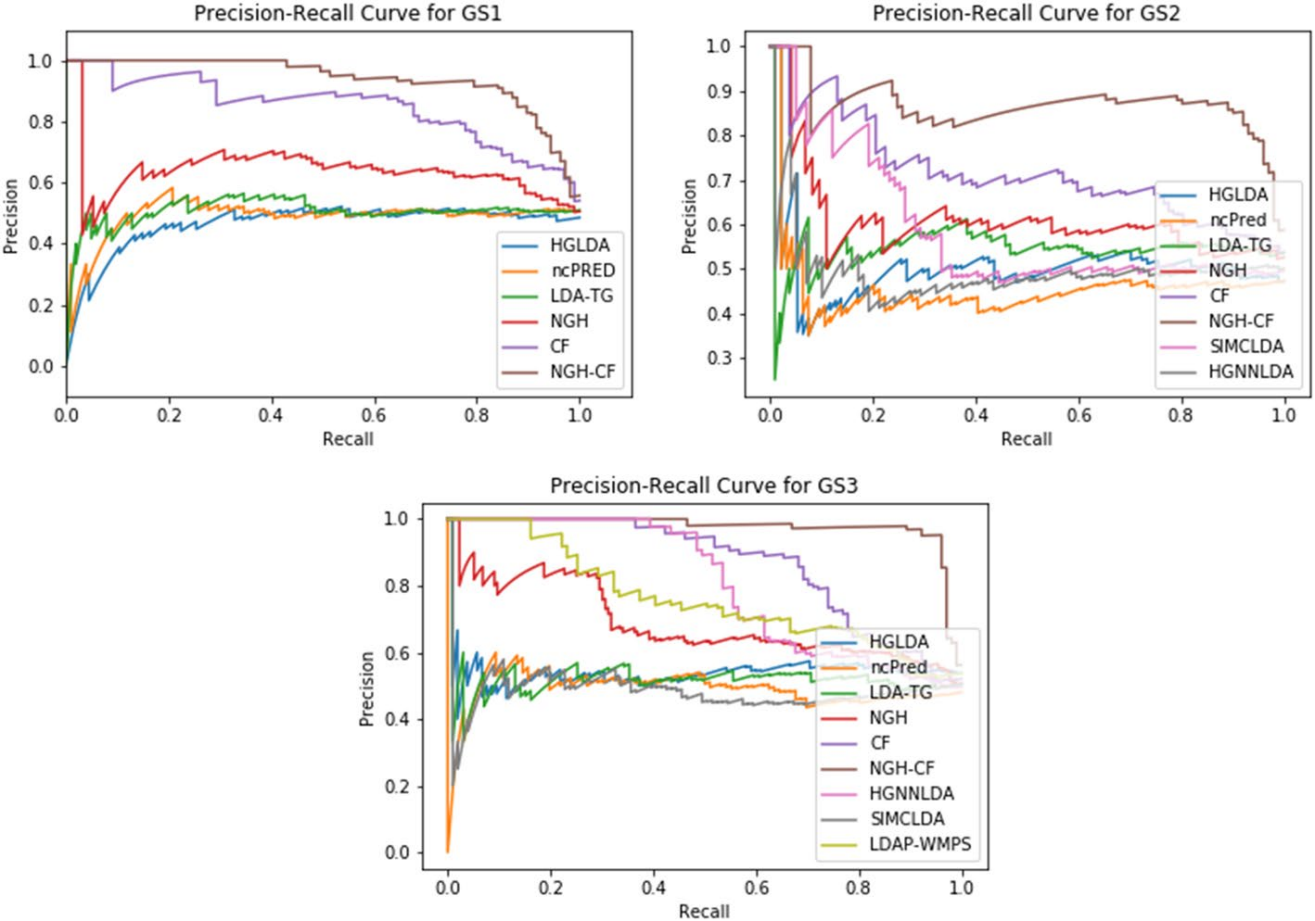
Precision-recall curves for the compared methods on GS1,GS2,GS3

The AUPR values scored by the compared methods on GS1, GS2, and GS3 are shown in Fig. 8, while the corresponding PR-curves are plotted in Fig. 9. In particular, for GS1 results are analogous to the ROC analysis, with NGH-CF the best performing one, followed by CF and NGH, while HGLDA is the worst. On GS2, NGH-CF and CF keep their superiority, followed by SMCLDA and NGH, while HGLDA is yet the worst one. On GS3, NGH-CF is the first, Cf the second and both HGNNLDA and LDAP-WMPS outperform NGH, while HGLDA in this case slightly outperforms LDA-TG, ncPred and SMCLDA, which results to be the worst one.

**Fig. 10:**
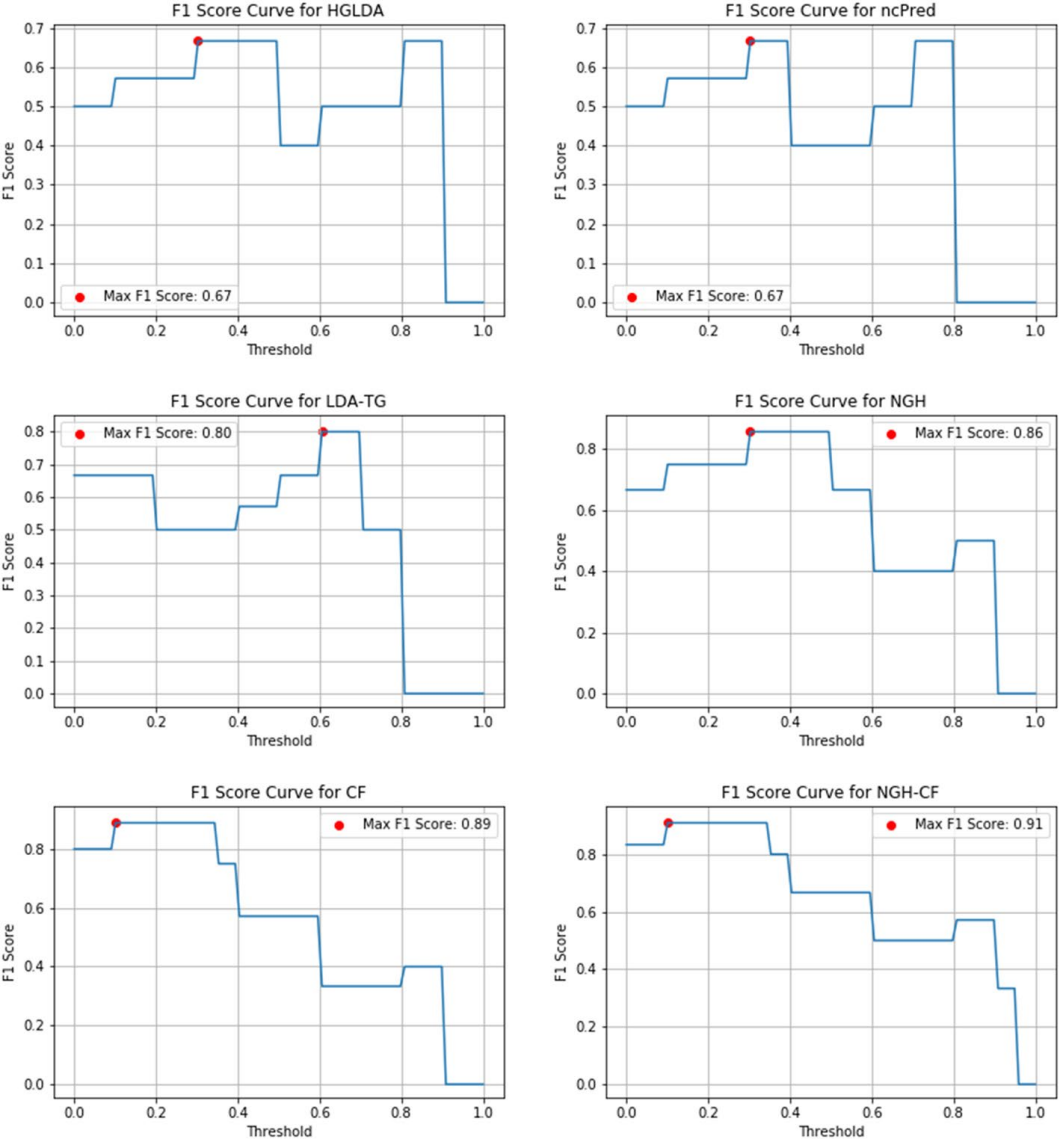
F1-score for the compared methods on GS1

**Fig. 11:**
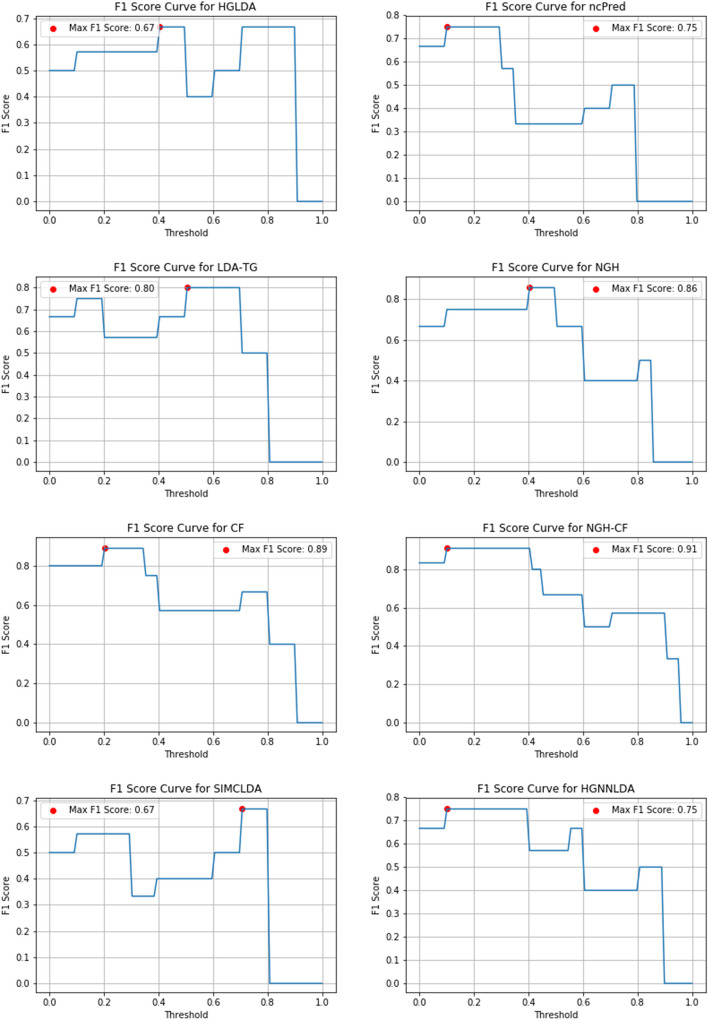
F1-Score for the compared methods on GS2

**Fig. 12:**
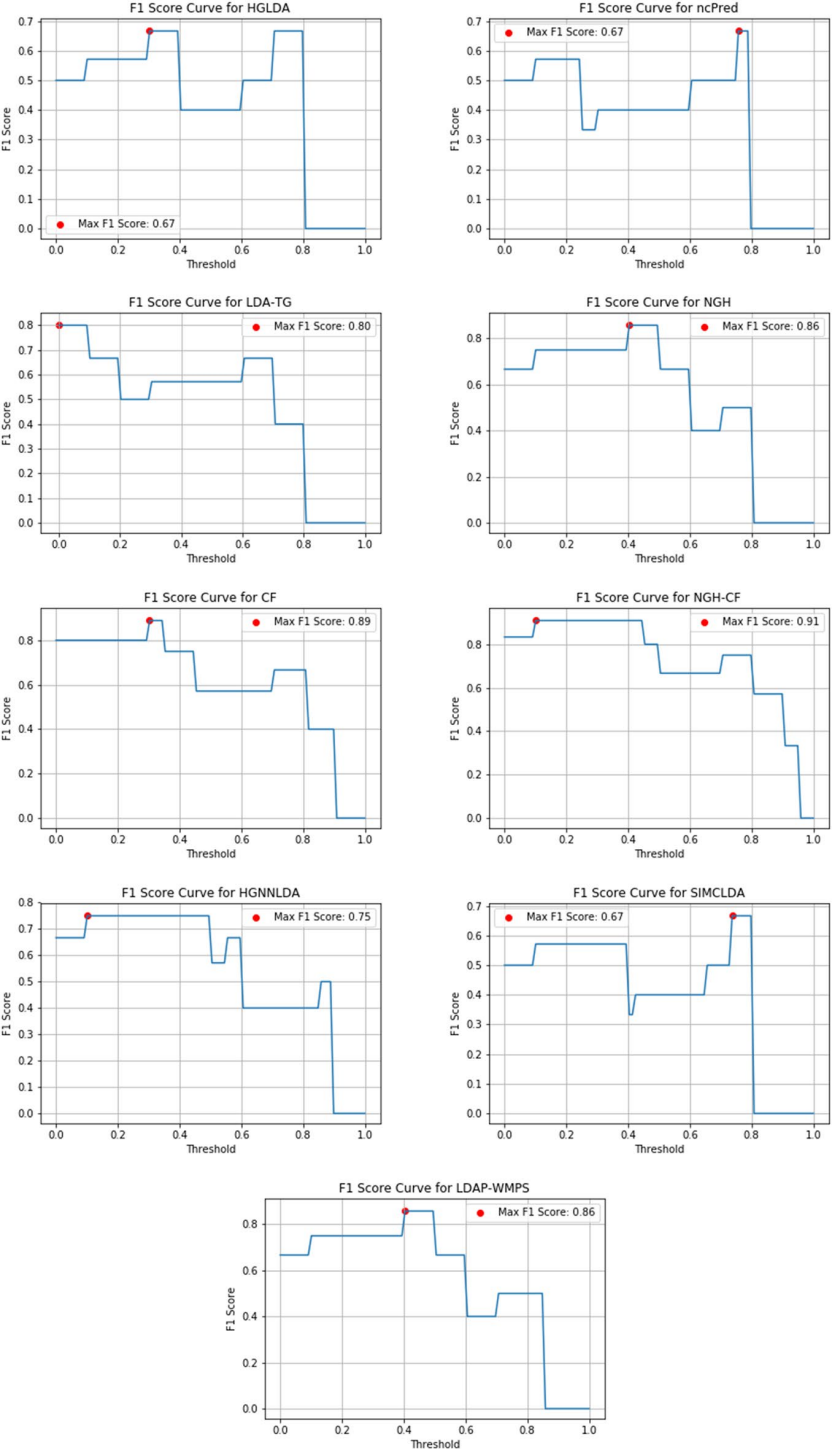
F1-Score for the compared methods on GS3

Figures 10, 11 and 12 show the F1-score values obtained, for all methods compared on GS1, GS2 and GS3, respectively, at the varying of a threshold fixed on the method score. In Tables 3, 4 and 5 it is shown, for each gold standard, the highest value of F1-score obtained by each considered method, as well as the corresponding Precision and Recall values, and the minimum threshold value for which the highest F1-score value has been reached. On GS1 and GS2, the three best performing approaches are NGH-CF, CF and NGH, in this order. On GS3 the order is the same, and LDAP-WMPS performs equally to NGH.

**Table 3:** F1-Score values (second column), corresponding precision and recall values (third and fourth columns, respectively), and corresponding threshold value of the score for each prediction method on GS1

GS1				
Method	Max F1-score	Precision	Recall	Threshold
HGLDA	0.67	0.48	0.98	0.30
ncPred	0.67	0.51	0.99	0.30
LDA-TG	0.80	0.50	0.99	0.60
NGH	0.86	0.51	0.99	0.30
CF	0.89	0.56	0.99	0.10
NGH-CF	0.91	0.56	0.99	0.10

**Table 4:** F1-Score values (second column), corresponding Precision and Recall values (third and fourth columns, respectively), and corresponding threshold value of the score for each prediction method on GS2

GS2				
Method	Max F1-score	Precision	Recall	Threshold
HGLDA	0.67	0.48	0.98	0.40
ncPred	0.75	0.51	0.98	0.10
LDA-TG	0.80	0.50	0.99	0.50
NGH	0.86	0.51	0.99	0.40
CF	0.89	0.56	0.99	0.20
NGH-CF	0.91	0.56	0.99	0.10
SIMCLDA	0.67	0.49	0.98	0.70
HGNNLDA	0.75	0.51	0.99	0.10

**Table 5:** F1-Score values (second column), corresponding Precision and Recall values (third and fourth columns, respectively), and corresponding threshold value of the score for each prediction method on GS3

GS3				
Method	Max F1-score	Precision	Recall	Threshold
HGLDA	0.67	0.49	0.98	0.30
ncPred	0.67	0.51	0.98	0.70
LDA-TG	0.80	0.51	0.99	0.40
NGH	0.86	0.51	0.99	0.40
CF	0.89	0.56	0.99	0.35
NGH-CF	0.91	0.56	0.99	0.10
SIMCLDA	0.67	0.49	0.98	0.75
HGNNLDA	0.75	0.51	0.99	0.10
LDAP-WMPS	0.86	0.50	0.98	0.4

### Robustness analysis

The main aim of the analysis discussed here is to measure to what extent the proposed methods are able to correctly recognize verified LDAs, even if part of the existing associations are missed, i.e., the sets of known and verified lncRNA-miRNA interactions and miRNA-disease associations are not complete. This is important to verify that the proposed approaches can provide reliable predictions also in presence of data incompleteness, that is often the case when lncRNAs are involved. Therefore, the robustness of each proposed method has been evaluated by performing progressive alterations of the input associations coming from the real datasets, according to the following three different criteria.

1. Progressively eliminate the $5\%$, $10\%$, $15\%$ and $20\%$ of lncRNA-miRNA interactions from the input data.
2. Progressively eliminate the $5\%$, $10\%$, $15\%$ and $20\%$ of miRNA-disease associations from the input data.
3. Progressively eliminate the $5\%$, $10\%$, $15\%$ and $20\%$ of both lncRNA-miRNA interactions and miRNA-disease associations (half and half), from the input data.

Tests summarized above have been performed for 20 times each. Tables 6, 7 and 8 show the mean of the AUC values for NGH, CF and NGH-CF, respectively, over the 20 tests. In particular, all methods perform well on the three test typologies at $5\%$, the worst being NGH-CF, which however presents an average AUC equal to 0.84 for case 1), that is still a high value. NGH-CF is also the method that presents the best robustness on case 3), keeping the value of 0.92 also at $20\%$, while CF is the worst performing in case 3), indeed its average AUC decreases from 0.95 at $5\%$ to 0.63 already at $10\%$, and then to 0.50 at $20\%$. This behaviour in case 3), where both lncRNA-miRNA interactions and miRNA-disease associations are progressively eliminated, deserves some observations. Indeed, results show that the combination of neighborhood analysis and collaborative filtering is the most robust one with regards to this perturbation, while collaborative filtering alone is the worst performing. On the other hand, CF results to be the most robust in case 1), where only lncRNA-miRNA interactions are eliminated, and this is due to the fact that CF does not take into account how many miRNAs are shared by pairs of lncRNAs. As for case 2), performance of all methods is comparable and generally good, possibly in consideration of the fact that a large number of miRNA-disease associations are available, therefore discarding small percentages of them does not affect largely the final prediction.

**Table 6:** The mean values of AUC scored by NGH over the 20 tests performed for each permutation case are shown

NGH	5%	10%	15%	20%
1	0.91	0.84	0.83	0.78
2	0.91	0.84	0.79	0.77
3	0.91	0.90	0.84	0.79

**Table 7:** The mean values of AUC scored by CF over the 20 tests performed for each permutation case are shown

CF	5%	10%	15%	20%
1	0.95	0.93	0.84	0.80
2	0.95	0.85	0.79	0.74
3	0.95	0.63	0.56	0.50

**Table 8:** The mean values of AUC scored by NGH-CF over the 20 tests performed for each permutation case are shown

NGH-CF	5%	10%	15%	20%
1	0.84	0.78	0.77	0.69
2	0.94	0.92	0.84	0.62
3	0.95	0.95	0.95	0.92

### Comparison on specific situations

In this section further experimental tests are described, showing how well the considered methods perform in detecting specific situations, depicted through the synthetic dataset first, and then searched for in the real data. In particular, the basic observation here is that prediction approaches from the literature usually fail in detecting true LDAs, when the involved lncRNAs and diseases do not have a large number of shared miRNAs (referring to those approaches taking the same input than ours). The novel approaches we propose are particularly effective in managing the situation depicted above, through neighborhood analysis and collaborative filtering, allowing to detect similar behaviours shared by different lncRNAs, depending on the miRNAs they interact with.

#### Synthetic data

For each set of LDAs defined in the synthetic data (i.e., set 1, set 2, and set 3), and for each tested method (i.e., HGLDA, NCPRED, NHG, CF, NGH-CF), Table 9 shows the percentage of LDAs in that set which is recognized at the top $10\%$, $20\%$, $30\%$, $50\%$ of the rank of all LDAs, sorted by the score returned by the considered method. As an example, for HGLDA the $32\%$ of LDAs of set 1 are located in the top $10\%$ of its rank, where instead none LDAs in set 2 or 3 find place.

Looking at these results some interesting considerations come out. First of all, for the methods HGLDA, NCPRED, NHG and CF most associations of the set 1 are located in the top $50\%$ of their corresponding ranks, while NGH-CF has a different behaviour. Indeed, it locates a lower number of such LDAs in the highest part of its rank than the other approaches, possibly due to the fact that it leaves room for a larger number of associations in the other two sets in the top ranked positions. As for LDAs in the set 2, all methods recognize some of them already in the top $10\%$, except for HGLDA, as alredy highlighted. The approaches able to recognize the larger percentages of these associations at the top $50\%$ of their rank are NGH and NGH-CF. LDAs in the set 3 are the most difficult to recognize, due to the fact that the lncRNA and the disease do not share any miRNA in common. Indeed, the worst performing methods in this case are HGLDA, which is able to locate some of these associations only at the top $50\%$ (according to the percentages we considered here), and NCPRED, which performs slightly better although it reaches the same percentage of located associations than HGLDA at $50\%$ (the $28\%$). As expected, approaches based on neighborhood analysis and collaborative filtering perform better, with the best one resulting to be NGH-CF.

#### Real data

In the previous section we have shown that all methods proposed here are able to detect specific situations, characterized by the fact that a lncRNA may have very few (or none) common miRNAs with a disease, and its neighbors share instead a large set of miRNAs with that disease. We have checked if this case occurs among the verified LDAs that our approaches find and their competitors do not. Table 10 shows, only by meaning of example, 10 experimentally verified LDAs, included in GS1, that are top ranked for the novel approaches proposed here, whereas they are in the bottom rank of the other approaches from the literature compared on GS1. Six out of such LDAs do not present any common miRNAs between the lncRNA and the disease, while four share only one miRNA. All involved lncRNAs present neighbors with a large number of miRNAs in common with the disease in that LDA, in accordance with the hypothesis that the ability in capturing this situation allows to obtain a better accuracy.

**Fig. 13:**
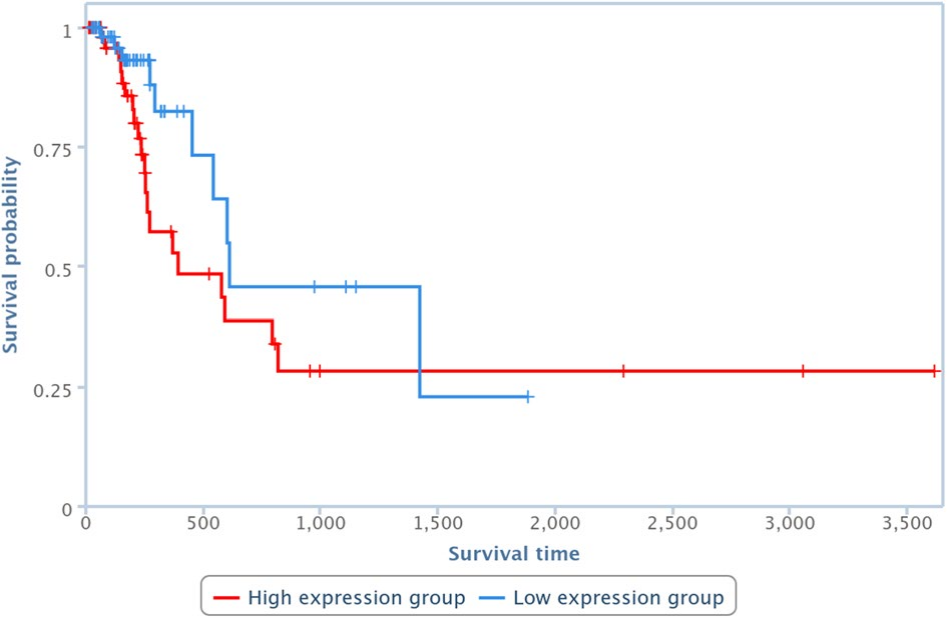
Survival analysis related to SNHG16 and bladder neoplasm

**Fig. 14:**
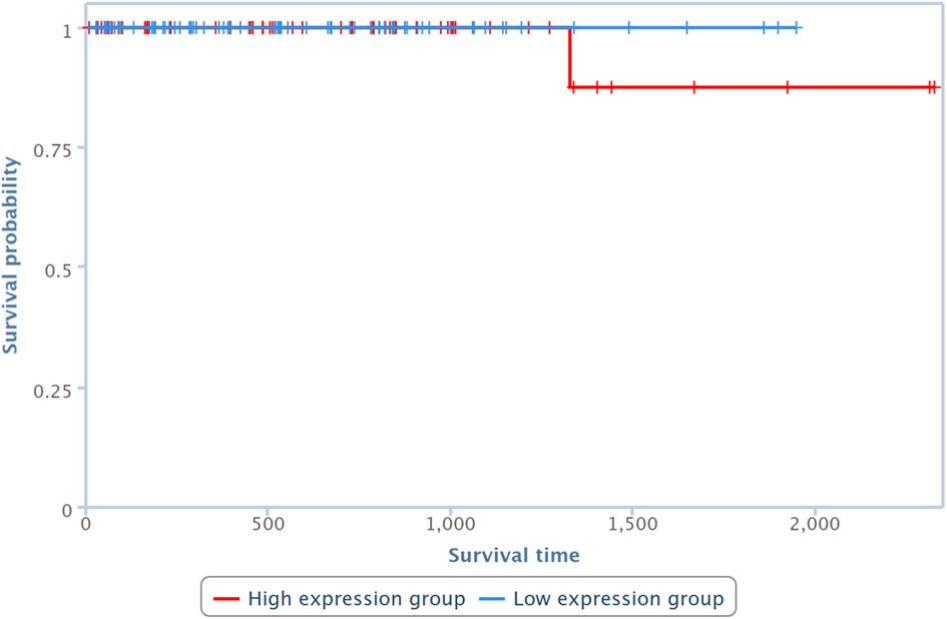
Survival analysis related to CBR3-AS1 and prostate neoplasm

**Fig. 15:**
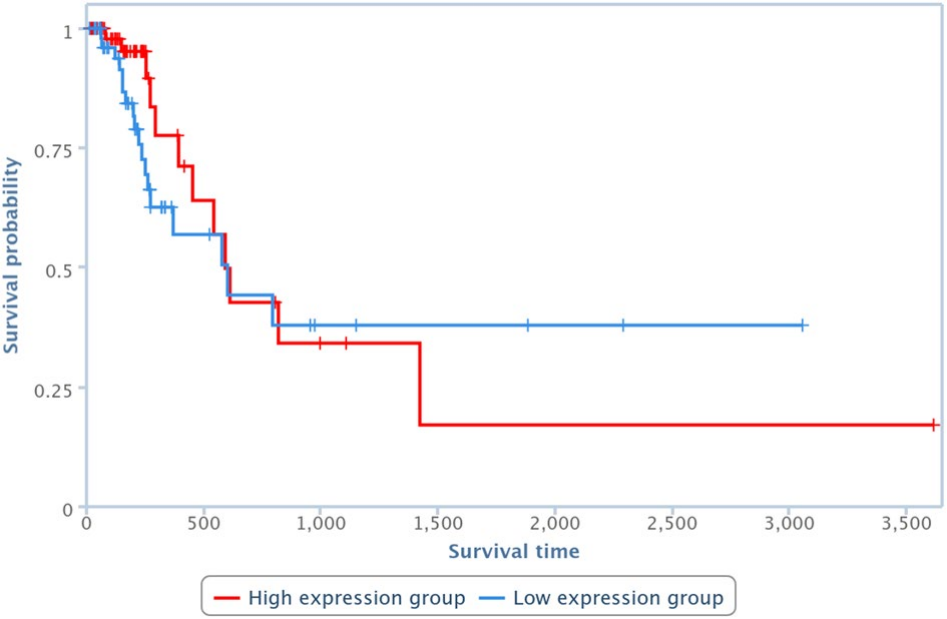
Survival analysis related to MALAT1 and bladder neoplasm

**Fig. 16:**
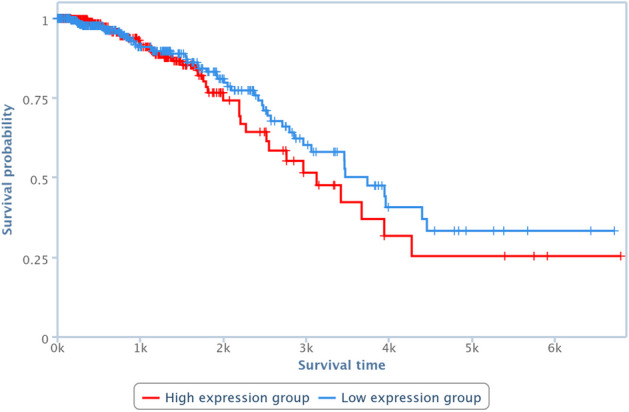
Survival analysis related to MEG3 and breast neoplasm

Survival analysis has been also performed by one of the TCGA Computational Tools, that is, TANRIC [41], on four of the pairs in Table 10. In particular, those lncRNAs and diseases available in TANRIC have been chosen. Results are reported in Figures 13, 14, 15 and 16, showing that the over-expression of the considered lncRNA determines a lower survival probability over the time, for all four considered cases.

**Table 9:** Percentage of LDAs in the set 1 (columns 2–5), set 2 (columns 6–9) and set 3 (columns 10–13) of the syntetic dataset, that has been recognized at the top $10\%$, $20\%$, $30\%$, $50\%$ of the rank obtained by sorting all LDAs (set 1, set 2, set 3 and others) according to the score, for each method

	Set 1				Set 2				Set 3			
Methods	10%	20%	30%	50%	10%	20%	30%	50%	10%	20%	30%	50%
HGLDA	0.32	0.56	0.84	0.96	0	0.13	0.19	0.5	0	0	0	0.28
NCPRED	0.24	0.52	0.68	0.80	0.13	0.19	0.37	0.62	0	0	0.07	0.28
LDATG	0.20	0.44	0.64	0.88	0.13	0.19	0.31	0.56	0.07	0.14	0.21	0.57
NGH	0.28	0.44	0.60	0.72	0.06	0.25	0.43	0.75	0	0.07	0.14	0.35
CF	0.20	0.40	0.56	0.72	0.13	0.25	0.43	0.56	0.07	0.14	0.21	0.57
NGH-CF	0.16	0.24	0.24	0.28	0.06	0.19	0.31	0.68	0.21	0.43	0.64	0.71

**Table 10:** LDAs of GS1 in the top rank of some novel method proposed here, and in the bottom rank of other approaches in the literature

LDA			Neighbours		Top rank percentile ( %)					
lncRNA	Disease	N. miRNAs	N. lncRNAs	N. miRNAs	HGLDA	NCPRED	LDATG	NGH	CF	NGH-CF
SNHG16	Bladder	0	64	65	95	90	80	5	10	11
	Neoplasm,									
CBR3-AS1	Prostate	0	7	12	70	69	50	10	6	5
	Neoplasm,									
SNHG3	Alzheimer	0	16	37	90	88	80	11	5	8
	Disease,									
CDKN2B-AS1	Cardiovascular	0	21	23	85	89	75	30	10	5
	Disease,									
CDKN2B-AS1	Diabetes	0	21	30	86	87	80	20	9	5
	Mellitus,									
CRNDE	Colorectal	0	147	149	90	95	88	15	5	8
	Neoplasm,									
MALAT1	Bladder	1	97	113	90	75	88	20	5	6
	Neoplasm,									
MEG3	Neoplasm	1	110	113	85	80	81	10	4	5
MEG3	Breast	1	200	205	80	55	60	22	6	5
	Neoplasm,									
MEG3	Lung	1	135	100	96	88	72	10	5	5
	Neoplasm,									

In the first three columns information on the LDA is reported: lncRNA, disease and the number of miRNA shared between them, respectively. Fourth and fifth columns show information on the neighbours of the lncRNA in the first column which share some miRNA with the disease in the second column. In particular, column 4 shows the number of such neighbours, while column 5 the number of miRNAs they share with the disease. Finally, the last six columns show the top percentile where the LDA is located in the rank of the considered method

### Discussion

In the previous sections the effectiveness and robustness of the proposed approaches have been illustrated, showing that all three are able to return reliable predictions, as well as to detect specific situations which may occur in true predictions and are missed by competitors. Here we provide a discussion on some novel LDAs predicted by NGH, CF and NGH-CF.

Table 11 shows seven LDAs which are not present in the considered gold standards, and that have been returned by all three methods proposed here, with highest score. The first of these associations is between CDKN2B-AS1 and LEUKEMIA, confirmed by recent literature [42, 43]. Indeed, CDKN2B-AS1 was found to be highly expressed in pediatric T-ALL peripheral blood mononuclear cells [42], moreover genome-wide association studies show that it is associated to Chronic Lymphocytic Leukaemia risk in Europeans [43]. As for the second association between DLEU2 and LEUKEMIA, DLEU2 is a long non-coding transcript with several splice variants, which has been identified by [44] through a comprehensive sequencing of a commonly deleted region in leukemia (i.e., the 13q14 region). Different investigations reported up regulation of this lncRNA in several types of cancers. The lncRNA H19 regulates GLIOMA angiogenesis [45, 46], while MAP3K14 is one of the well-recognized biomarkers in the prognosis of renal cancer, which is reminiscent of the pancreatic metastasis from renal cell carcinoma [47]. MEG3 has been recently found to be important for the prediction of LEUKEMIA risk [48]. Multiple studies have shown that MIR155HG is highly expressed in diffuse large B-cell (DLBC) lymphoma and primary mediastinal B-cell lymphoma, and in chronic lymphocytic leukemia. The transcription factor MYB activates MIR155HG activity, which causes the epigenetic state of MIR155HG to be dysregulated and causes an abnormal increase in MIR155 [49]. Also the last top-ranked association in Table 11 between TUG1 and NON-SMALL CELL LUNG CARCINOMA has found evidence in the literature [50–52].

**Table 11:** LDAs with high score according to all presented methods and that are not in the gold standard (the prediction score value is reported in the last three columns, respectively)

LncRNA	Disease	NGH	CF	NGH-CF
CDKN2B-AS1	LEUKEMIA	0.95	0.96	0.97
DLEU2	LEUKEMIA	0.91	0.92	0.93
H19	GLIOMA	0.88	0.90	0.91
MAP3K14	PANCREATIC NEOPLASMS	0.83	0.84	0.84
MEG3	LEUKEMIA	0.80	0.83	0.82
MIR155HG	LEUKEMIA, B-CELL	0.79	0.82	0.81
TUG1	CARCINOMA, NON-SMALL-CELL LUNG	0.76	0.77	0.78

Tables 12, 13, and 14 show the top 100 (sorted by the scores returned by each method) novel LDA predictions that NGH and CF, NGH and NGH-CF, CF and NGH-CF have in common, respectively. Many of the lncRNAs involved in such top-ranked LDAs are not yet characterized in the literature, therefore results presented here may be considered a first attempt to provide novel knowledge about them, through their inferred association with known diseases.

**Table 12:** First 100 novel LDAs predicted as the consensus between NGH and CF

Consensus between CF and NGH			
lncRNA	Disease	CF Score	NGH score
AC005152.2	PARKINSON DISEASE	0.99	0.99
AC007566.10	CARCINOMA, ENDOMETRIOID	0.99	0.98
AC015849.16	DEMYELINATING DISEASES	0.99	0.98
BZRAP1-AS1	CENTRAL NERVOUS SYSTEM DISEASES	0.99	0.97
CTB-89H12.4	UTERINE CERVICAL NEOPLASMS	0.99	0.97
CTC-550B14.6	GRAVES DISEASE	0.99	0.97
FLI1-AS1	CHORDOMA	0.99	0.96
H19	MYOTONIC DYSTROPHY	0.99	0.96
HCG18	HYPERTENSION	0.99	0.96
KCNQ1OT1	DIGESTIVE SYSTEM NEOPLASMS	0.99	0.96
KIAA1984-AS1	ASTHMA	0.99	0.96
LIFR-AS1	DIABETIC NEPHROPATHIES	0.99	0.96
LINC00661	ENDOMETRIOSIS	0.99	0.95
LINC00667	LEUKEMIA, MYELOID	0.99	0.95
LINC00667	LEUKEMIA, MYELOID	0.99	0.95
MEG8	UTERINE CERVICAL NEOPLASMS	0.99	0.95
RP11-102F4.3	LYMPHOMA, MANTLE-CELL	0.99	0.94
RP11-108P20.1	FIBROBLASTS	0.99	0.94
RP11-108P20.1	RENAL INSUFFICIENCY	0.99	0.93
RP11-159D12.9	URINARY BLADDER NEOPLASMS	0.99	0.93
RP11-169K16.9	PROSTATIC NEOPLASMS	0.99	0.93
RP11-174G17.2	NASAL POLYPS	0.99	0.93
RP11-184E9.2	FRANCISELLA	0.99	0.93
RP11-216F19.2	CARCINOMA, HEPATOCELLULAR	0.99	0.92
RP11-221J22.2	DEMYELINATING DISEASES	0.99	0.92
RP11-429D19.1	HEART FAILURE	0.99	0.92
RP11-618G20.1	MUSCULAR DYSTROPHY, DUCHENNE	0.99	0.92
RP11-67L3.4	HCV	0.99	0.92
RP6-24A23.7	MELANOMA	0.99	0.92
SPPL2B	LEUKEMIA, LYMPHOCYTIC, CHRONIC, B-CELL	0.99	0.92
RP11-365O16.6	BLADDER NEOPLASMS	0.98	0.92
RP11-379K17.11	LEUKEMIA, LYMPHOCYTIC, CHRONIC, B-CELL	0.98	0.92
RP11-767N6.7	CEREBRAL ISCHEMIA	0.98	0.91
MAL2	ENDOMETRIOSIS	0.98	0.91
RP11-797A18.6	LEUKEMIA, BIPHENOTYPIC, ACUTE	0.98	0.91
RP4-665N4.8	SCLERODERMA, LOCALIZED	0.98	0.90
SCAMP1	BREAST NEOPLASMS	0.98	0.90
SCGB1B2P	CARCINOMA, SQUAMOUS CELL	0.98	0.89
TTTY15	MYELODYSPLASTIC SYNDROMES	0.98	0.89
MIR3179-1	HCV	0.98	0.89
MIR3179-1	CEREBRAL ISCHEMIA	0.98	0.89
AC005152.2	SCHIZOPHRENIA	0.98	0.88
AC007036.5	CICATRIX	0.98	0.88
AC007255.7	LEIOMYOSARCOMA	0.98	0.88
AC084219.4	MYELODYSPLASTIC SYNDROMES	0.98	0.88
C1RL-AS1	COLORECTAL NEOPLASMS	0.98	0.88
C1RL-AS1	COLORECTAL NEOPLASMS	0.98	0.87
CTBP1-AS1	MELANOMA	0.98	0.87
CTC-338M12.2	PEMPHIGUS, BENIGN FAMILIAL	0.98	0.87
FBXL19-AS1	URINARY BLADDER NEOPLASMS	0.98	0.87
FLI1-AS1	AMYOTROPHIC LATERAL SCLEROSIS	0.98	0.87
LEMD1-AS1	FRANCISELLA	0.98	0.87
LINC00707	LUNG NEOPLASMS	0.98	0.86
RP11-1055B8.4	MYOCYTES, CARDIAC	0.98	0.86
RP11-105N14.1	ABORTION, HABITUAL	0.98	0.86
RP11-123K3.4	OLIGODENDROGLIOMA	0.98	0.86
RP11-139H15.1	HEMANGIOSARCOMA	0.98	0.85
RP11-184E9.2	ENCEPHALOMYELITIS, AUTOIMMUNE, EXPERIMENTAL	0.98	0.85
RP11-184E9.2	COLORECTAL NEOPLASMS, HEREDITARY NONPOLYPOSIS	0.98	0.85
RP11-277L2.2	RETINAL NEOVASCULARIZATION	0.98	0.84
RP11-290D2.4	LEUKEMIA, MYELOID	0.98	0.84
RP11-290F20.1	MULTIPLE SCLEROSIS	0.98	0.84
RP11-290F20.3	PULMONARY EMBOLISM	0.98	0.83
AC005083.1	STOMACH NEOPLASMS	0.97	0.83
AC025171.1	CARCINOMA, DUCTAL, BREAST	0.97	0.83
ALMS1-IT1	PRRSV INFECTION	0.97	0.83
C11ORF95	HEART FAILURE	0.97	0.83
COX10-AS1	TOXOPLASMOSIS	0.97	0.83
CTA-204B4.6	GASTROINTESTINAL NEOPLASMS	0.97	0.83
CTC-338M12.2	NASAL POLYPS	0.97	0.83
CTC-459F4.3	HEPATITIS C	0.97	0.83
CTC-487M23.5	PULMONARY DISEASE, CHRONIC OBSTRUCTIVE	0.97	0.82
H19	CARCINOMA, HEPATOCELLULAR	0.97	0.82
HOTAIR	LEUKEMIA, B-CELL	0.97	0.82
LEMD1-AS1	GASTRITIS, ATROPHIC	0.97	0.81
LEMD1-AS1	GRAFT VS HOST DISEASE	0.97	0.81
LIFR-AS1	PARKINSON DISEASE	0.97	0.80
MATN1-AS1	DIABETIC RETINOPATHY	0.97	0.80
MIAT	RHABDOMYOSARCOMA	0.97	0.80
MIR3179-1	LIVER CIRRHOSIS, BILIARY	0.97	0.80
MIR4720	NASOPHARYNGEAL NEOPLASMS	0.97	0.80
RP11-108P20.1	ENCEPHALOMYELITIS, AUTOIMMUNE, EXPERIMENTAL	0.97	0.80
RP11-108P20.1	INTERVERTEBRAL DISK	0.97	0.79
RP11-184E9.2	CYSTIC FIBROSIS	0.97	0.79
RP11-184E9.2	INTERVERTEBRAL DISK	0.97	0.79
RP11-184E9.2	GASTRITIS, ATROPHIC	0.97	0.79
RP11-184E9.2	FIBROBLASTS	0.97	0.79
RP11-203J24.9	CARDIOMYOPATHIES	0.97	0.79
RP11-206L10.11	NEUROBLASTOMA	0.97	0.79
RP11-264L1.3	NASAL POLYPS	0.97	0.79
RP11-277P12.20	LIPOSARCOMA	0.97	0.79
RP11-290F20.3	CENTRAL NERVOUS SYSTEM DISEASES	0.97	0.79
RP11-331F9.10	AMYOTROPHIC LATERAL SCLEROSIS	0.97	0.78
RP11-344B2.2	ASTHMA	0.97	0.78
RP11-355O1.11	LEUKEMIA, B-CELL	0.97	0.78
RP11-355O1.11	TOXOPLASMOSIS	0.97	0.78
RP11-429J17.7	DIABETIC RETINOPATHY	0.97	0.78
RP3-523K23.2	NEOPLASMS, SQUAMOUS CELL	0.97	0.77
RP4-659J6.2	PHEOCHROMOCYTOMA	0.97	0.77
AC005532.5	PEMPHIGUS, BENIGN FAMILIAL	0.96	0.77

**Table 13:** First 100 novel LDAs predicted as the consensus between NGH and NGH-CF

Consensus between NGH and NGH-CF			
lncRNA	Disease	NGH score	NGH-CF score
SLC26A4-AS1	KIDNEY DISEASES	1.0	0.88
RP11-44F14.11	SARCOMA	1.0	0.88
VPS11	HIV	1.0	0.88
RP11-380L11.4	VASCULAR DISEASES	1.0	0.88
RP11-367N14.2	LYMPHOMA	1.0	0.87
RNU12	NEOPLASMS	1.0	0.87
RP11-37B2.1	GLIOMA	1.0	0.87
RP11-77H9.2	SARCOMA	1.0	0.87
RP11-221J22.2	GLOMERULONEPHRITIS	1.0	0.86
SLC26A4-AS1	SARCOMA	1.0	0.86
SNHG1	PERIODONTITIS	1.0	0.86
RP11-361F15.2	CERVICAL NEOPLASMS	1.0	0.86
RP11-305N23.1	HEPATITIS	1.0	0.85
RP3-523K23.2	RECTAL NEOPLASMS	1.0	0.85
RP11-618G20.1	SARCOMA	1.0	0.84
RP11-277P12.20	SARCOMA	1.0	0.84
RP1-59M18.2	LEUKEMIA	1.0	0.84
RP11-819C21.1	HEPATITIS	1.0	0.83
RP11-175K6.1	SARCOMA	1.0	0.83
RP11-390P2.4	SARCOMA	1.0	0.83
RP11-68L18.1	HEPATITIS	1.0	0.83
RP11-983P16.4	SARCOMA	1.0	0.83
RP11-206L10.11	FIBROSIS	1.0	0.83
SCARNA10	ADENOMA	1.0	0.83
PDXDC2P	SARCOMA	1.0	0.83
RP11-66N24.4	SARCOMA	1.0	0.83
RP11-214C8.5	ADENOMA	1.0	0.83
RP11-158K1.3	FIBROSIS	1.0	0.82
RP11-2C24.4	SARCOMA	1.0	0.82
RP11-221J22.1	SARCOMA	1.0	0.82
RP5-886K2.3	LEUKEMIA	1.0	0.82
RP11-457M11.2	SARCOMA	1.0	0.82
RP11-701H24.4	ECLAMPSIA	1.0	0.80
RP5-1172N10.3	SARCOMA	1.0	0.80
RP11-690D19.3	BLADDER NEOPLASMS	1.0	0.80
RP11-498E2.8	LEUKEMIA	1.0	0.80
RP11-797A18.6	LEUKEMIA	1.0	0.79
RP11-690D19.3	LUNG DISEASES	1.0	0.79
RP11-126O1.5	CARCINOMA	1.0	0.79
RP5-1028K7.2	SARCOMA	1.0	0.79
RP11-849I19.1	ADENOMA	1.0	0.79
RP11-399K21.11	KIDNEY DISEASES	1.0	0.79
SCAMP1	HEPATITIS	1.0	0.79
RP11-496I9.1	DEMENTIA	1.0	0.78
RP11-54O7.1	EYE ABNORMALITIES	1.0	0.78
RP11-108P20.1	FIBROSIS	1.0	0.78
RP11-492E3.1	LUNG DISEASES	1.0	0.78
RP11-677M14.3	LEUKEMIA, MYELOID	1.0	0.78
RP11-761E20.1	LYMPHOMA	1.0	0.78
RP11-293M10.6	SARCOMA	1.0	0.77
RP11-310H4.2	HEPATITIS	1.0	0.77
XXBAC-BPG254F23.6	DIABETES MELLITUS	1.0	0.77
PVT1	SARCOMA	1.0	0.77
RP11-403I13.8	LEUKEMIA	1.0	0.77
RP11-996F15.2	SARCOMA	1.0	0.77
RP11-478C19.2	COLITIS	1.0	0.77
RP11-252P19.3	NEOPLASMS	1.0	0.77
RP11-473I1.10	GLOMERULONEPHRITIS	1.0	0.77
RP11-54O7.1	VASCULAR DISEASES	1.0	0.77
RP11-24B19.4	HEPATITIS	1.0	0.76
RP11-213H15.3	CARCINOMA	1.0	0.76
SNHG1	BLADDER NEOPLASMS	1.0	0.76
RP11-529K1.2	LEUKEMIA	1.0	0.76
RP11-24B19.4	SARCOMA	1.0	0.76
RP11-506M13.3	CERVICAL NEOPLASMS	1.0	0.76
RP11-480D4.3	HEPATITIS	1.0	0.76
RP11-498D10.6	DEMENTIA	1.0	0.75
RP11-98I9.4	CARCINOMA	1.0	0.75
RP11-521C20.4	ADENOMA	1.0	0.75
RP5-1024G6.5	KIDNEY DISEASES	1.0	0.75
U47924.19	RECTAL NEOPLASMS	1.0	0.74
RP11-379K17.4	GLOMERULONEPHRITIS	1.0	0.73
RP11-690G19.3	SARCOMA	1.0	0.73
WDR7-UA1	NERVOUS SYSTEM DISEASES	1.0	0.73
RP11-324L3.3	LEUKEMIA	1.0	0.73
SNHG1	MOYAMOYA DISEASE	1.0	0.72
RP11-284N8.3	ARTHRITIS	1.0	0.72
ST8SIA6-AS1	LUNG DISEASES	1.0	0.72
RP11-178G16.4	NEOPLASMS	1.0	0.72
SNHG1	LIPOSARCOMA	1.0	0.72
RP11-421L21.3	SARCOMA	1.0	0.71
RP11-649G15.2	LEUKEMIA	1.0	0.71
RP11-363E7.4	SARCOMA	1.0	0.71
RP11-344B2.2	NEOPLASMS	1.0	0.70
RP11-24B19.4	LEUKEMIA	1.0	0.70
RP11-492E3.1	ADENOMA	1.0	0.69
RP11-290F20.1	LEUKEMIA	1.0	0.69
RP11-119F7.5	LEUKEMIA	1.0	0.68
RP11-761E20.1	NEOPLASMS	1.0	0.68
RP11-498C9.15	SARCOMA	1.0	0.67
RP11-160O5.1	NEOPLASMS	1.0	0.67
RP11-418J17.1	RECTAL NEOPLASMS	1.0	0.67
RP11-767N6.7	ISCHEMIA	1.0	0.67
RP11-758M4.4	FIBROSIS	1.0	0.67
RP11-50E11.3	RECTAL NEOPLASMS	1.0	0.67
RP11-403I13.8	LEUKEMIA	1.0	0.66
RP5-886K2.3	SARCOMA	1.0	0.65
RP11-571M6.8	NEOPLASMS	1.0	0.65
RP11-54O7.1	MUSCULAR DYSTROPHY, DUCHENNE	1.0	0.64
RP5-1172N10.3	KIDNEY DISEASES	1.0	0.64

**Table 14:** First 100 novel LDAs predicted as the consensus between CF and NGH-CF

Consensus between NGH and NGH-CF			
lncRNA	Disease	CF score	NGH-CF score
RP11-132A1.3	SCLERODERMA, SYSTEMIC	0.88	0.88
RP11-123K3.4	LEIOMYOSARCOMA	0.87	0.88
RP11-330L19.4	GRAFT VS HOST DISEASE	0.87	0.88
RECQL4	ABORTION, HABITUAL	0.87	0.88
PDXDC2P	CERVICAL INTRAEPITHELIAL NEOPLASIA	0.87	0.87
RP11-357C3.3	DIABETIC RETINOPATHY	0.87	0.87
RP11-227G15.3	PRRSV INFECTION	0.87	0.87
RP11-315H15.2	ISCHEMIA	0.86	0.87
RP11-193H5.1	PAPILARY THYROID CARCINOMA	0.86	0.86
RP11-105N14.1	LIVER CIRRHOSIS, BILIARY	0.86	0.86
RP11-229P13.25	CRYPTOSPORIDIUM	0.86	0.86
RP11-1094M14.11	PAIN	0.86	0.86
RP11-119F19.2	NASAL POLYPS	0.85	0.85
RP11-286H14.6	CYSTIC FIBROSIS	0.85	0.85
RP11-393M11.2	MOUTH NEOPLASMS	0.85	0.84
RP11-344B2.2	BRAIN INJURIES	0.85	0.84
RP11-227D2.3	LYMPHOMA	0.85	0.84
RP11-140H17.1	GRAVES DISEASE	0.84	0.83
RP11-214K3.21	AZOOSPERMIA	0.84	0.83
PVT1	PULMONARY EMBOLISM	0.84	0.83
RP11-141O11.2	LYMPHOMA, MANTLE-CELL	0.83	0.83
RP11-464F9.1	HCV	0.83	0.83
RP11-355O1.11	FIBROSIS	0.83	0.83
RP11-27I1.2	PITUITARY ADENOMAS	0.83	0.83
RP11-267N12.3	PROLACTINOMA	0.83	0.83
RP11-153A23.6	CHOLANGIOCARCINOMA	0.83	0.83
RP11-290D2.4	MYOTONIC DYSTROPHY	0.83	0.83
RNU12	FANCONI ANEMIA	0.83	0.82
RP11-425M5.5	ARTHRITIS, PSORIATIC	0.83	0.82
RP11-307E17.8	MULTIPLE SCLEROSIS	0.83	0.82
RP11-298I3.1	PSYCHOTIC DISORDERS	0.82	0.82
RP11-244H3.1	CERVICAL INTRAEPITHELIAL NEOPLASIA	0.82	0.82
RP11-35G9.3	CREUTZFELDT-JAKOB SYNDROME	0.82	0.80
RP11-261C10.5	MUSCULAR DYSTROPHIES	0.82	0.80
RP11-2C24.4	CHOLESTEATOMA	0.82	0.80
RP11-443N24.2	FANCONI ANEMIA	0.81	0.80
RP11-304M2.2	EYE ABNORMALITIES	0.81	0.79
RP11-11N9.4	AORTIC ANEURYSM, ABDOMINAL	0.80	0.79
RP11-284M14.1	CATARACT	0.80	0.79
RP11-244O19.1	MOUTH NEOPLASMS	0.80	0.79
RP11-174G17.2	ANXIETY DISORDERS	0.80	0.79
RP11-473I1.9	DIABETIC NEPHROPATHIES	0.80	0.79
RP11-421L21.3	FRAGILE X SYNDROME	0.80	0.79
RP11-418J17.3	OSTEOARTHRITIS	0.80	0.78
RP11-383J24.5	PULMONARY FIBROSIS	0.79	0.78
PDXDC2P	ENDOMETRIAL NEOPLASMS	0.79	0.78
RP11-304M2.2	DIABETIC NEPHROPATHIES	0.79	0.78
RP11-276H19.1	MYOCARDITIS	0.79	0.78
RP11-380L11.4	BRAIN INJURY	0.79	0.78
RP11-44F14.11	BRAIN INJURY	0.79	0.77
PRIM2	CEREBRAL INFARCTION	0.79	0.77
PTOV1-AS1	HAND, FOOT AND MOUTH DISEASE	0.79	0.77
RP11-140H17.1	PROSTATE NEOPLASMS	0.79	0.77
RP11-1186N24.5	ATRIAL FIBRILLATION	0.79	0.77
RP11-379H18.1	BRAIN INJURY	0.79	0.77
RP11-396C23.2	PHEOCHROMOCYTOMA	0.79	0.77
RP11-290D2.4	ANXIETY DISORDERS	0.78	0.77
RP11-145M9.4	CARDIOMEGALY	0.78	0.77
RP11-221J22.2	PROSTATIC NEOPLASMS	0.78	0.77
RP11-203J24.9	ACQUIRED IMMUNODEFICIENCY SYNDROME	0.78	0.76
RP11-392P7.6	ADRENOCORTICAL ADENOMA	0.78	0.76
RP11-276H19.2	RHABDOMYOSARCOMA	0.78	0.76
RP11-220I1.1	ARTHRITIS, RHEUMATOID	0.77	0.76
RP11-214K3.21	DRUG-INDUCED LIVER INJURY	0.77	0.76
RP11-159F24.1	SCHIZOPHRENIA	0.77	0.76
RP11-461L13.3	ADENOMA	0.77	0.76
RP11-261C10.5	ESOPHAGUS	0.77	0.75
RP11-20G6.3	CARCINOMA, BASAL CELL	0.77	0.75
RP11-109M17.2	MYELOPROLIFERATIVE DISORDERS	0.77	0.75
RP11-154J22.1	FRAGILE X SYNDROME	0.77	0.75
RP11-429J17.7	HEMANGIOMA	0.77	0.74
RP11-252P19.3	ACUTE CORONARY SYNDROME	0.77	0.73
RP11-155D18.12	GASTRITIS, ATROPHIC	0.77	0.73
RP11-277L2.2	MUSCULAR DYSTROPHY, FACIOSCAPULOHUMERAL	0.76	0.73
RP11-169D4.1	HUNTINGTON DISEASE	0.76	0.73
RP11-18F14.2	NASAL POLYPS	0.76	0.72
RP11-121C2.2	PRECURSOR T-CELL LYMPHOBLASTIC LEUKEMIA-LYMPHOMA	0.76	0.72
RP11-324L3.3	HEPATITIS C, CHRONIC	0.76	0.72
RP11-213H15.3	HIV-1	0.76	0.72
RP11-475D8.1	ASTROCYTOMA	0.76	0.72
RP11-384K6.6	HEMANGIOSARCOMA	0.75	0.71
RP11-325D5.3	ADENOMA	0.75	0.71
RP11-405O10.2	LEIOMYOMA	0.75	0.71
RP11-458D21.1	FIBROBLASTS	0.75	0.70
RP11-303E16.2	SARS VIRUS	0.74	0.70
RP11-16B13.1	ABORTION, HABITUAL	0.74	0.69
RP11-174G6.5	FRONTOTEMPORAL LOBAR DEGENERATION	0.73	0.69
RP11-118N24.3	CENTRAL NERVOUS SYSTEM DISEASES	0.73	0.68
RP11-446J8.1	MUSCULAR DYSTROPHY, FACIOSCAPULOHUMERAL	0.73	0.68
RP11-133N21.10	SCLERODERMA, SYSTEMIC	0.73	0.67
RP11-160E2.6	PERIODONTITIS	0.73	0.67
PRR7-AS1	UTERINE CERVICAL NEOPLASMS	0.72	0.67
RP11-392P7.6	SARCOMA, EWING’S	0.72	0.67
RP11-380L11.4	INFLAMMATORY BOWEL DISEASES	0.72	0.67
PRR7-AS1	TOXOPLASMOSIS	0.72	0.67
RP11-140H17.1	BLADDER NEOPLASMS	0.72	0.66
RP11-436A20.4	COLON NEOPLASMS	0.72	0.65
RP11-286H14.6	DIABETES MELLITUS	0.72	0.65
RP11-173M1.8	ADRENOCORTICAL ADENOMA	0.71	0.64
PRKAG2-AS1	ESOPHAGEAL NEOPLASMS	0.71	0.64

## Conclusion

We have explored the application of neighborhood analysis, combined with collaborative filtering, for the improvement of LDAs prediction accuracy. The three approaches proposed here have been evaluated and compared first against their direct competitors from the literature, i.e., the other methods which also use lncRNA-miRNA interactions and miRNA-disease associations, without exploiting a priori known LDAs. It results that all methods proposed here are able to outperform direct competitors, the best one (NGH-CF) also significantly (AUC equal to 0.966 against the 0.886 by NCPRED). In particular, it has been shown that the improvement in accuracy is due to the fact that our approaches capture specific situations neglected by competitors, relying on similar lncRNAs behaviour in terms of their interactions with the considered intermediate molecules (i.e., miRNAs). The proposed approaches have been then compared also against other recent methods, taking different inputs (e.g., integrative approaches), and the experimental evaluation shows that they are able to outperform them as well.

It is worth pointing out the importance of providing reliable data in input to the LDAs prediction approaches. As discussed in this manuscript, information on the lncRNAs relationships with other molecules, and between intermediate molecules and diseases, is provided in input to the proposed approaches. Reliable datasets have been used to perform the experimental analysis provided here. However, as the user may provide also different input datasets, it is important to point out that the reliability of the obtained predictions strictly depends on that of input information.

As neighborhood analysis has resulted to be effective in characterizing lncRNAs with regards to their association with known diseases, we plan to apply it also for predicting possible common functions among lncRNAs, for example by clustering them according to their interactions, which has shown to be successful for other types of molecules [53]. Moreover, due to the success of integrative approaches on the analysis of biological data [54], we expect that including other types of intermediate molecules, such as for example genes and proteins, in the main pipeline of the proposed approaches may further improve their accuracy.

In conclusion, the use of reliable input data and the integration of different types of information coming from molecular interactions seem to be the most promising future directions for LDAs prediction.

## Data Availability

The source code is available at: https://github.com/marybonomo/LDAsPredictionApproaches.git In particular, executable software for NGH, CF, and NGH-CF are provided, as well as syntetic and real input datasets used here; the three different gold standard datasets GS1, GS2, GS3; the final obtained results.
